# The divergent roles of tryptophans W354 and W217 in OCT1 transport: Similar localization, distinct functions

**DOI:** 10.1016/j.jbc.2025.111051

**Published:** 2025-12-13

**Authors:** Sarah Römer, Lennart Ebel, Anna Neumann, Vincent Rönnpagel, Lukas Schulig, Marleen J. Meyer-Tönnies, Mladen V. Tzvetkov

**Affiliations:** 1 Department of General Pharmacology, Institute of Pharmacology, Center of Drug Absorption and Transport (C_DAT), University Medicine Greifswald, Greifswald, Germany; 2 Department of Pharmaceutical and Medicinal Chemistry, Institute of Pharmacy, University Greifswald, Greifswald, Germany

**Keywords:** polyspecificity, promiscuity, organic cation transporter 1, OCT1, SLC22A1, membrane transport, structure-function, transporter, drug transport

## Abstract

Hepatic organic cation transporter 1 (OCT1, *SLC22A1*) has a broad spectrum of structurally diverse substrates, including drugs like morphine, metformin, sumatriptan, and fenoterol. Here, we leveraged existing cryo-EM data to identify amino acids that constrain the binding pocket toward the intracellular lumen and performed functional analyses to determine their roles in OCT1 transport, in general, and in substrate specificity, in particular. We mutated W217, W354, and I446 and analyzed their effects on the transport of 27 structurally highly diverse substrates in HEK293 cells. The W354A mutant resulted in a complete loss of function for all tested substrates, whereas only the W354Y mutant partially retained OCT1 activity. Molecular dynamics simulations and functional analyses suggest that W354 affects OCT1 transport by forming a hydrogen bond with N453 in transmembrane helix 10, thereby stabilizing OCT1 in its outward open conformation. W217A showed strongly substrate-dependent effects, with the aromatic ring of tryptophan being the key property affecting transport. Smaller, less lipophilic substrates were more strongly affected. The substrate-specific effects of the W217A mutant correlated significantly with those of the F244A mutant. The double mutant W217A/F244A strongly reduced uptake of all tested substrates, indicating a compensatory mechanism between these two amino acids. I446 showed only marginal effects on OCT1 transport. Our data suggest that W354 is crucial for OCT1 function by mediating the switch from an inward-occluded to inward-open conformation but is not directly involved in substrate interaction. In contrast, W217, along with the previously known F244, contributes to the substrate specificity of OCT1.

Organic cation transporter 1 (OCT1, *SLC22A1*) is primarily expressed in the liver, where it is localized in the sinusoidal membrane of hepatocytes (1, 2, 3, 4). This localization makes OCT1 an important determinant in the pharmacokinetics of organic cationic drugs, influencing whether they remain in the systemic circulation or are taken up into the liver for metabolism. As such, OCT1 represents a potential target for drug–drug interactions (DDIs) that can impact both pharmacokinetics and drug efficacy (5, 6, 7). Moreover, OCT1 is of clinical relevance due to its high genetic variability. Common OCT1 polymorphisms have been reported to affect the pharmacokinetics of various drugs, including fenoterol, sumatriptan, metformin, and proguanil (8, 9, 10, 11).

Already with its initial cloning in 1994, OCT1 was described as a polyspecific transporter capable of recognizing a wide variety of chemically diverse substrates (12). Currently, more than 200 drugs have been identified as OCT1 substrates (13, 14, 15, 16). OCT1 transport is not limited to drugs but also includes endogenous compounds such as thiamine and serotonin (17, 18). Typically, OCT1 substrates are weak bases or tertiary amines, all of which are positively charged at physiological pH. The molecular weight of substrates transported by OCT1 generally ranges from 100 to 500 Da (13, 15). From a structural perspective, OCT1 accommodates a highly diverse substrate spectrum—from small molecules like tetraethylammonium (TEA^+^) or metformin to bulky and complex compounds such as methylnaltrexone or ipratropium (Fig. S1). Due to this broad substrate range, OCT1 is often referred to as a promiscuous transporter, emphasizing the lack of strict substrate specificity (19, 20, 21). However, studies on morphinan opioids with highly similar structures have shown substantially different inhibitory potencies, suggesting that OCT1 does exhibit ligand selectivity (22). Moreover, analyses of substrate uptake in other drug classes—such as β2-adrenergic agonists and tetraalkylammonium compounds—indicate that even minor structural modifications can significantly influence a compound's ability to be transported by OCT1 (23, 24).

More than 30 years after the first description of OCT1, the precise mechanisms underlying its broad yet selective substrate recognition and transport remain incompletely understood. Initial assumptions suggested that the positively charged substrates are always bound by a single negatively charged residue within the binding pocket—such as D474 (25) or E386 (26). However, these assumptions have been strongly challenged by recently resolved cryo-EM structures and accompanying functional data suggesting a more variable interaction of the positive charge of the substrate (19, 24, 27). A key element in the transport mechanism, like the YER motif—comprising Y361, E386, and R439—has been identified and functionally validated (24, 27). Furthermore, for most structurally resolved substrates, transport appears to be orchestrated through interactions with multiple, primarily aromatic residues (19, 26, 27, 28, 29, 30, 31, 32). Among these, F244 was recently shown to contribute to transport in a substrate-dependent manner, highlighting its role in the polyspecific nature of OCT1 (20).

Multiple amino acids clearly contribute to the formation of the substrate-binding pocket (Fig. 1*A*). However, their distribution within the pocket differs, suggesting potential differences in their roles in the transport process. While the YER motif is involved in closing the outer gate of the binding pocket, W217, F244, W354, and I446 line, among others, the bottom of the pocket in the outward-open conformation (Fig. 1*A*) and close it toward the intracellular lumen (Fig. S2). Unfortunately, the limited number of substrates resolved by cryo-EM leaves a gap in our understanding of their precise roles in OCT1 transport and substrate specificity.

**Figure 1:**
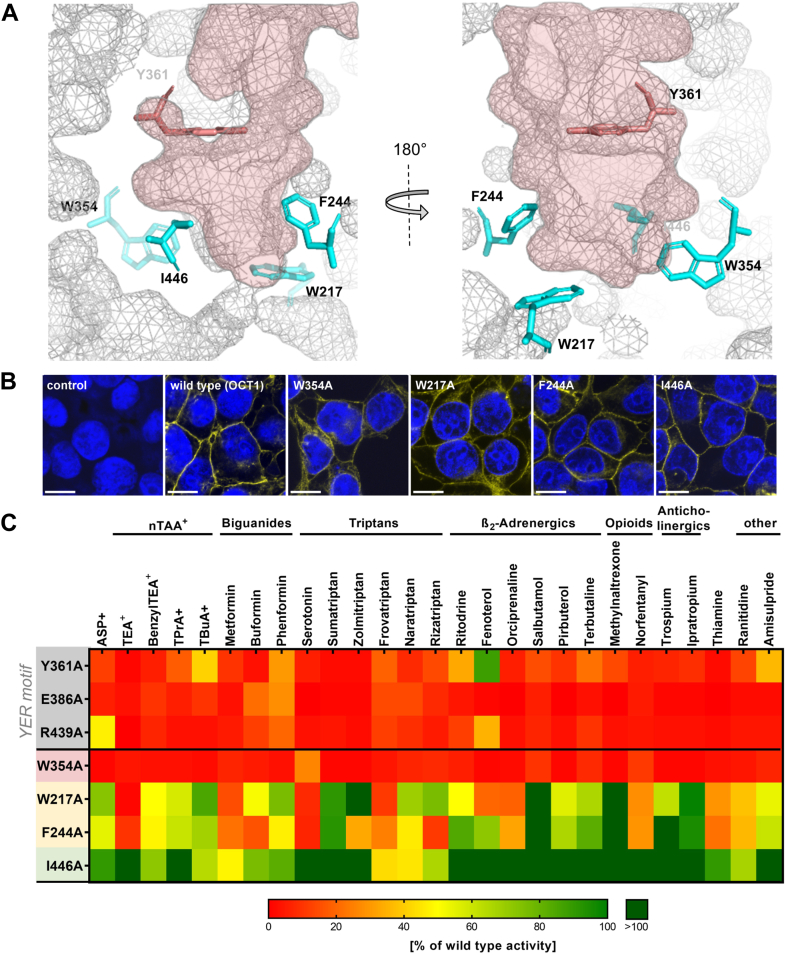
**Effects of alanine mutants of amino acids that form the substrate-binding pocket**. *A*, schematic representation of the aromatic residues W217, F244, and W354, as well as the nonpolar I446 (all in *cyan*) that contribute into the formation of the OCT1 substrate-binding pocket (represented as mesh, *red*) and were suggested by cryo-EM to be directly involved in substrate interaction. Y361 (*red*) has already been reported to be strongly involved in formation of the binding pocket and mediating transport (24) OCT1 is shown in outward-occluded conformation (PDB: 8JTT), from the side with the top of the protein facing the extracellular space (27). *B*, verification of membrane localization of alanine mutants in stably transfected HEK293 cells; scale bar represents 10 μm. *C*, effects of the mutants on the uptake of 27 selected OCT1 substrates. The uptake was measured at single concentrations (concentrations used are listed in Table S1) into HEK293 cells, stably overexpressing the mutants; active uptake was normalized to wild type activity after subtraction of passive diffusion into empty vector control cells. Shown are the means of n = 3 to 5 independent experiments. The newly analyzed amino acids were ordered from strong (*top*) to limited effects (*bottom*) on OCT1 transport. Data on the effects within the YER domain were partially reported before (24) but are shown here for comparison. OCT1, organic cation transporter 1.

Two tryptophan residues—W217 and W354—were proposed to be involved in substrate transport based on initial mutagenesis studies and homology models (29, 33, 34), and their role was later confirmed by recent cryo-EM structures (19, 26, 27). In addition, I446, a nonaromatic but nonpolar residue located within the binding pocket, has been highlighted in some cryo-EM structures as potentially involved in substrate transport (26).

This study aims to investigate the roles of the amino acids W217, W354, and I446 in the overall transport process and substrate selectivity. To this end, we mutated each of these amino acids to alanine and analyzed the impact on membrane localization and transport function. When alanine mutants exhibited strong effects, we analyzed further additional mutations to other amino acids to identify key features essential for transport. Finally, we employed molecular dynamics (MD) simulations to support the functional experiments.

## Results

### Divergent effects of W217A, W354A, and I446A mutants on OCT1 transport

First, we analyzed the general role of W217, W354, and I446 in OCT1 transport by mutating each individually to alanine and testing the effects on a broad range of OCT1 substrates (Fig. 1). As controls and for comparison of transport effects, we included other key amino acids in the binding pocket that have been previously studied—namely the YER motif (Y361, E386, R439; (24)) and F244 (20). The generated mutants were stably transfected into HEK293 cells, and their correct membrane localization was confirmed (Fig. 1*B*). All mutants were clearly localized in the plasma membrane except for the W354A mutant, which was only partially localized in the membrane (Fig. 1*B*). We analyzed the effects of alanine mutation on the transport capabilities by measuring the transport of 27 known OCT1 substrates covering a structurally highly diverse substrate spectrum at single concentrations (Fig. 1*C*).

The W354A mutant completely abolished the transport of any substrate tested. Thereby, the effects of the W354A mutant on transport were similar to those observed for the YER motif (E386A, R439A, and partially also Y361A), which was previously demonstrated to be crucial for the transport mechanism. The substrate-wide loss of activity for the W354A mutant may only be partially explained by suboptimal membrane localization.

In contrast to the W354A mutant, the W217A mutant affected the substrate uptake in a substrate-dependent manner. Among the 27 tested substrates, the W217A mutant reduced uptake of nine substrates to less than 50% of wild type activity. Among these affected substrates were not only small substrates like TEA^+^ and metformin but also large, complex structures like fenoterol or frovatriptan. Thereby, the observed substrate-specific effects of the W217A mutant were similar to those of the F244A mutant.

The I446A mutant had only limited effects on the uptake. I446A did not affect OCT1 transport of 21 out of 27 tested substrates. The maximal reduction was observed by frovatriptan (reduction of uptake by 57% compared to the wild type). Indeed, for some substrates like salbutamol and terbutaline, the uptake was even increased (by 300% and 336%, respectively). Therefore, the I446 residue was not further analyzed.

### Role of W354 in the conformational switch of OCT1

**Figure 2:**
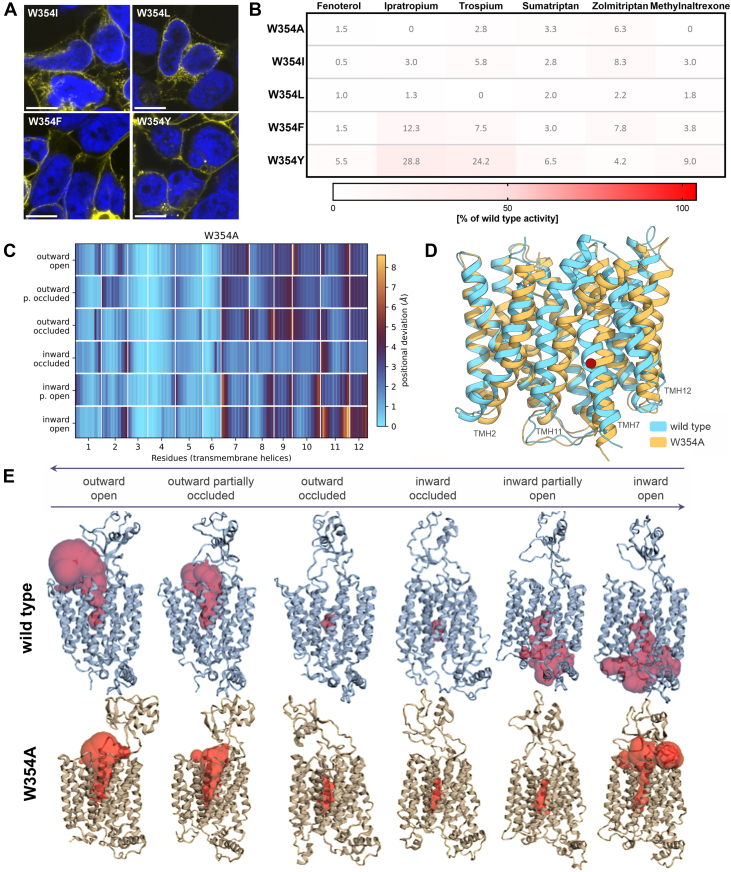
**The role of the side chain at codon 354**. Different W354 mutants were transiently transfected in HEK293 cells, and their ability to transport selected OCT1 substrates was analyzed. *A*, verification of membrane localization of W354 mutants ; scale bar represents 10 μm. *B*, uptake of key OCT1 substrates; active uptake was normalized to wild type activity after subtraction of passive diffusion into empty vector control cells. Concentrations used are listed in Table S1. Shown are means of n = 4 to 6 independent experiments. *C*, stability of the W354A mutant across different conformational states. Per-residue Cα RMSD (transmembrane helices) of the W354A mutant compared to the wild type structure across all conformational states. Mutant structures were generated by averaging the last 500 snapshots from three independent molecular dynamics (MD) simulations. The W354 mutant (located in TMH7) induces significant perturbations, notably affecting surrounding transmembrane helices due to structural collapse and rearrangements. *D*, structural alignment of the wild type (*blue*) and W354A (*orange*) mutant in the inward-open state exemplarily displays substantial conformational changes, particularly in the C-terminal region. Residue 354 located in TMH7 is indicated by a *red* sphere. *E*, comparison of the wild type (*upper* panel) and the W354A mutant (*lower* panel) in terms of protein conformation and substrate-binding pocket (*red*) across all conformational states. OCT1 is shown from the side with the top of the protein facing the extracellular space. OCT1, organic cation transporter 1; TMH, transmembrane helix; MD, molecular dynamics.

We analyzed the specific role of the side chain at amino acid residue 354 for transport. Therefore, we additionally mutated W354 to isoleucine (W354I) and leucine (W354L) to introduce a comparable hydrophobic side chain without an aromatic ring. Similar to the alanine mutation, both mutants were only partially expressed in the plasma membrane and were unable to transport any of the tested substrates (Fig. 2, *A* and *B*, Fig. S3). When mutating to phenylalanine (W354F) to introduce an aromatic side chain, the correct membrane localization was restored, but transport was abolished for most of the substrates. W354F showed only limited uptake of trospium and ipratropium (8% and 15% of wild type activity, respectively).

Finally, we mutated W354 to tyrosine (W354Y) to simultaneously introduce an aromatic feature and a hydrogen bond donor also present in tryptophan. The W354Y mutant showed the highest retainment of OCT1 activity. However, it was maximally 25% to 30% of wild type activity for trospium and ipratropium, respectively, while it remained inactive for all other substrates tested.

This suggests a key role of W354 in OCT1 transport, which includes both the presence of an aromatic ring and hydrogen bond interaction, as well as the specific nature of the side chain. However, this role probably does not involve a direct interaction with a substrate.

Following the wet-lab experiments, we performed MD simulations to investigate the conformational changes of the W354A and W354F mutants and to propose possible mechanisms underlying their near-complete loss of function. Based on cryo-EM structures that captured all key conformational states of the transport cycle (27), we first modeled the complete transition process for the wild type protein (Fig. S4). These models then served as the basis for MD simulations of the W354A and W354F mutants.

The W354A mutant caused substantial conformational changes within the C-terminal domain (transmembrane helices (TMH)7 to 12) across all simulated conformational states (Fig. 2*C*). Especially in the inward-open conformation, the positions of TMH7, 11, and 12 within the transporter were strongly affected (Fig. 2*D*). Most importantly, the introduction of the alanine mutant prevented the complete opening of the substrate-binding pocket toward the intracellular lumen (Fig. 2*E*). The mutant rather switched somewhat back to partially outward-open conformation, with its pocket opened toward the extracellular space but not toward the cytoplasm. This observation explains the lack of measurable intracellular accumulation of any of the tested substrates. Similarly, retaining an aromatic ring in the W354F mutant strongly affected the stability of the C-terminal domain and opening of the binding pocket toward the cytoplasm (Fig. S5). Taken together, the MD simulations suggest that W354 plays an essential role in the opening of the protein toward the intracellular space, thereby explaining the observed loss of activity with all tested substrates.

### Interaction partners of W354

**Figure 3:**
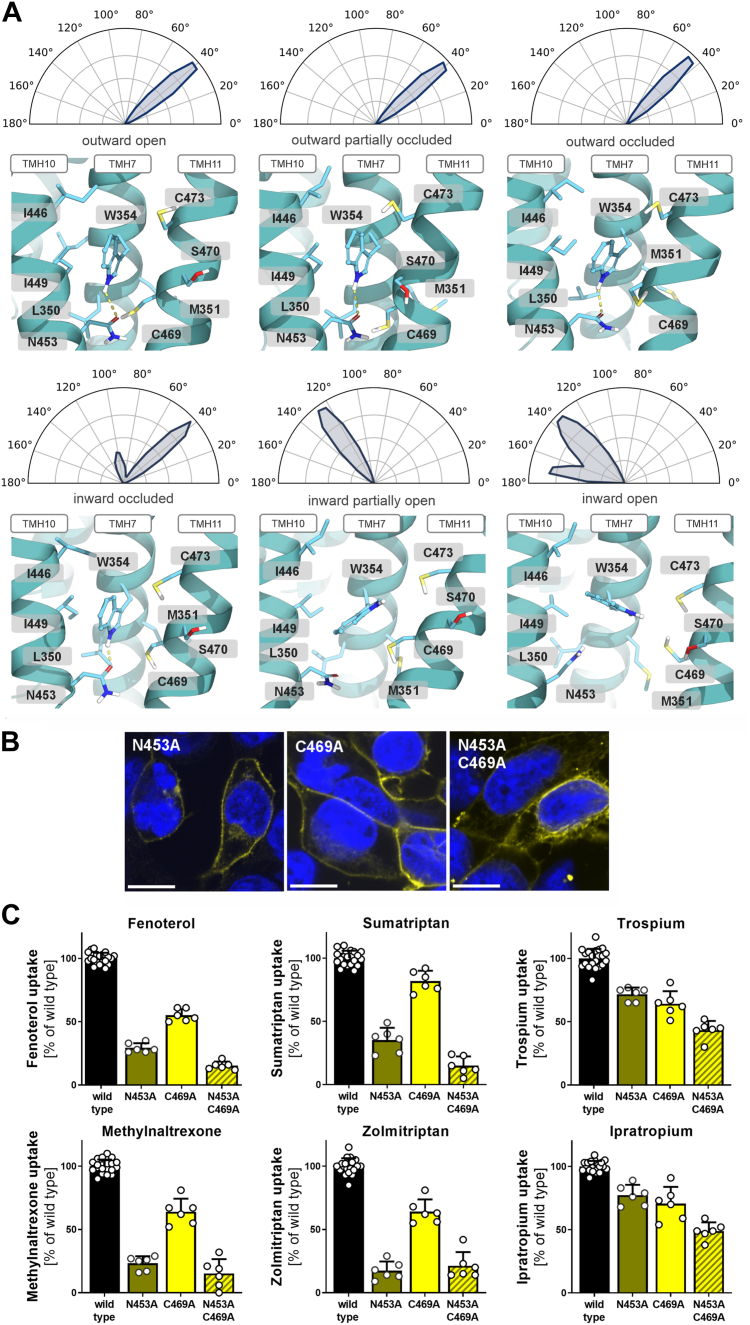
**Molecular dynamics and experimental data supporting the role of N453 as the functionally relevant interaction partner of W354**. *A*, interactions and local environment of W354 across different conformational states of OCT1 modeled in MD simulations. Polar plots show the normalized angle distribution between the backbone and the indole ring of W354, calculated from 20,000 snapshots per state obtained from MD simulations. In the outward-open state, W354 is tightly packed between TMH10 and TMH11, forming a stable hydrogen bond with N453. As the protein transitions toward the inward-open state, the surrounding space expands, disrupting the tight packing and weakening the hydrogen bond interaction with N453. OCT1 is shown from the side, with the viewpoint centered on the substrate-binding pocket facing the TMH7/10/11 interface, and the top of the protein oriented toward the extracellular space. *B*, verification of membrane localization of mutants in transiently transfected HEK293 cells; scale bar represents 10 μm. *C*, uptake of key OCT1 substrates into HEK293 cells transiently transfected with alanine mutants of N453 or C469 or a double mutant of N453A and C469A; active uptake was normalized to wild type activity after subtraction of passive diffusion into empty vector control cells; concentrations used are listed in Table S1; shown are means ± SD of n = 6 independent experiments. OCT1, organic cation transporter 1; MD, molecular dynamics; TMH, transmembrane helix.

We analyzed potential interactions of W354 with neighboring residues. In outward-open conformation, W354, as part of TMH7, is anchored between TMH10 and TMH11 (Fig. 3*A*). Potential interaction partners are I446 and N453 from TMH10 and C469 from TMH11. We employed computational and wet-lab approaches to analyze these interactions.

On one hand, we used MD simulations of the entire translocation process to assess the role of W354 as an interaction partner in the wild type. The simulations emphasized a hydrogen bond between W354 and N453. This interaction was maintained from outward-open toward inward-occluded but was disrupted during the transition from inward-occluded toward inward-open states. Upon disruption of this hydrogen bond, a free rotation of the indole ring of W354 and an expansion of space between TMHs 7, 10, and 11 was observed (Fig. 3*A*).

In parallel, we analyzed potential interactions of W354 in wet-lab experiments. The I446A mutant was included in the initial screening and did not affect transport (Fig. 1). Therefore, it was excluded, and we focused on N453 and C469 as potential interaction partners of W354. Both N453A and C469A mutants were expressed correctly in the plasma membrane (Fig. 3*B*). C469A showed only limited effects on transport, with uptake of fenoterol being most strongly affected, albeit with a maximal reduction of transport by 53% (Fig. 3*C*). In contrast, N453A exhibited strong but substrate-dependent effects on transport. Uptake of fenoterol, zolmitriptan, and methylnaltrexone was strongly reduced (ranging from 19% to 33% of the wild type activity). In contrast, uptake of trospium and ipratropium was comparable to the wild type (70% to 82% of the wild type activity).

The double mutant N453A-C469A did not result in a substantial reduction in transport compared to the N453A single mutant. The observed 43 to 65% decrease in trospium and ipratropium transport (Fig. 3*C*) may be attributed to reduced membrane localization of the double mutant (Fig. 3*B*). Thus, both MD simulations and experimental data indicate that N453 is the primary interaction partner of W354.

### An aromatic ring rather than a hydrogen bond donor of W217 is important for substrate interaction

**Figure 4:**
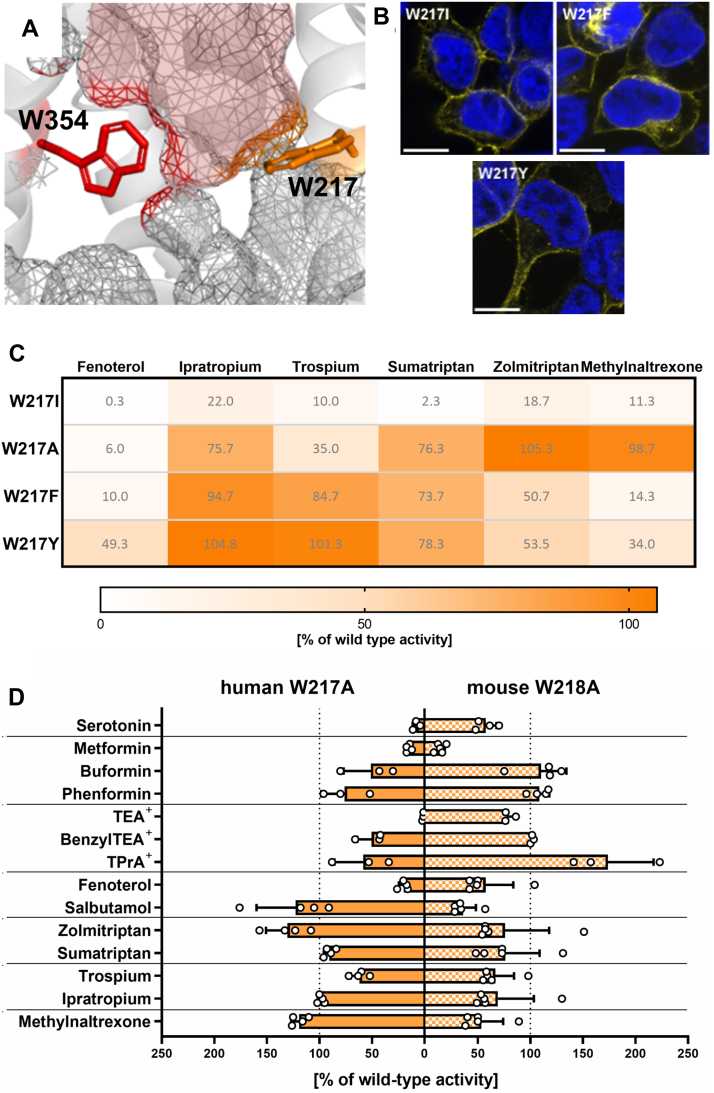
**Role of the side chain at codon 217 for OCT1 function**. *A*, localization of W217 in comparison to W354 within the binding pocket in outward-open state; PDB: 8JTS (27) OCT1 is shown from the side with the top of the protein facing the extracellular space. OCT1-binding pocket is indicated as *red* mesh. *B*, verification of membrane localization of mutants in transiently transfected HEK293 cells; scale bar represents 10 μm. *C*, uptake of key OCT1 substrates into HEK293 cells transiently transfected with different mutants of W217. *D*, comparison of uptake activity between human and murine W217A/W218A mutants; active uptake was normalized to wild type activity after subtraction of passive diffusion into empty vector control cells; concentrations used are listed in Table S1; shown are means ± SD of n = 3 to 5 independent experiments. OCT1, organic cation transporter 1.

Similarly to W354, W217 constrains the basal barrier of the binding pocket (Fig. 4*A*). However, in contrast to W354, the W217A mutant did not completely abolish transport but rather affected substrate uptake in a strongly substrate-dependent manner (Fig. 1*C*). Similar to W354, we extended the W217 mutation spectrum to amino acids covering different functionalities of tryptophan. All mutants were located in the plasma membrane (Fig. 4*B*).

The mutations resulted in highly diverse, substrate-dependent effects on transport activity. The uptake of fenoterol, which was strongly dependent on W217 (Figs. 1 and 4*C*), was only partially retained (49% of the wild type) by the W217Y mutant. For trospium uptake, the pattern was similar but with wild type activity of both the W217F and the W217Y mutant (Figs. 4*C* and S6). In contrast, the uptake of sumatriptan and ipratropium was not or only weakly dependent on the W217 residue (except for the W217I mutant). This did not change when W217 was mutated to other aromatic amino acids. Interestingly, methylnaltrexone uptake was not affected by the W217A mutant but was strongly reduced when another aromatic amino acid was introduced at codon 217. For zolmitriptan, the effects were similar but less pronounced (Figs. 4*C* and S6).

W217I was the only mutant that caused a reduction of more than 75% in the uptake of all tested substrates (Figs. 4*C* and S6) despite the correct membrane localization (Fig. 4*B*). One possible explanation is that the hydrophobicity of the W217I mutant exerts an artifactual effect on protein structure, leading to a loss of function without pointing to a precise mechanism for W217 itself.

Furthermore, we extended our analysis of W217 effects to the mouse Oct1 ortholog. The substrate-dependent effects of W217A were much more prominent in human than in mouse OCT1. In contrast to human OCT1, the murine W218A mutant (corresponding to W217A in human OCT1) affected substrate uptake to a lower extent (Figs. 4*D*, S7). Among fourteen tested substrates, only metformin and salbutamol were transported to less than 50% of wild type activity by the murine W218A mutant. More importantly, the murine W218A mutant only marginally reduced the uptake of TEA^+^, serotonin, and fenoterol (retaining 80%, 58%, and 57% of wild type activity, respectively). The uptake of each of these substrates was reduced to less than 10% of the wild type activity in the corresponding human mutant.

### Substrate features defining sensitivity to W217

**Figure 5:**
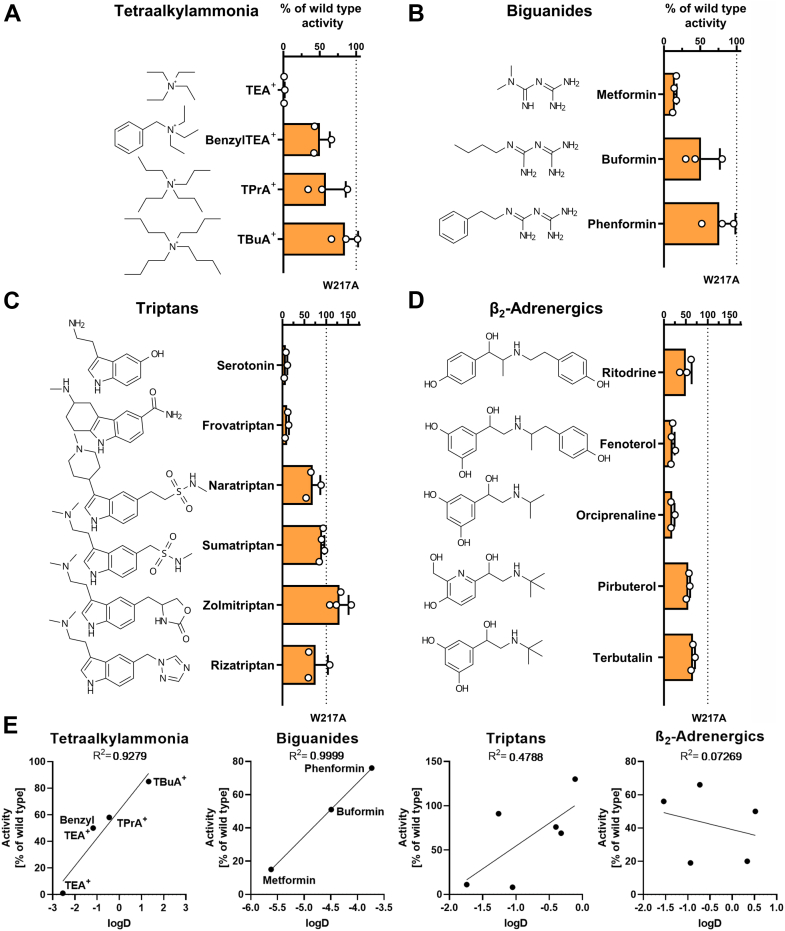
**Influence of ligand structure on uptake by the W217A mutant**. Uptake of tetraalkylammonia (*A*), biguanides (*B*), triptans (*C*), and β2-adrenergics (*D*) was analyzed in HEK293 cells stably overexpressing the W217A mutant; active uptake was normalized to wild type activity after subtraction of passive diffusion into empty vector control cells; concentrations used are listed in Table S1; shown are means ± SD of n = 3 to 4 independent experiments. *E*, correlation between the substrate-specific logD values and the uptake activity observed in the W217A mutant. Shown are means of n = 3 to 4 independent experiments. OCT1, organic cation transporter 1.

Finally, we analyzed the effects of W217 in the context of minor structural variations in the substrate. To this end, we measured the uptake of the W217A mutant using groups of similar drugs, such as triptans, β2-adrenergics, and biguanides, as well as model substrates, including tetraalkylammonium (nTAA) compounds (Fig. 5). When analyzing the uptake of different nTAA, the uptake of TEA^+^ (the smallest nTAA transported by OCT1) was abolished in the W217A mutant (Fig. 5*A*). However, uptake of the W217A mutant increased with increasing lipophilicity of the substrate, as shown by introducing an aromatic ring (BenzylTEA^+^) or longer lipophilic side chains (TPrA^+^, TBuA^+^), reaching up to 85% of the wild type activity when TBuA^+^ was used (Fig. 5, A and *E*, S8). Similarly, when analyzing biguanides, the uptake of the W217A mutant was strongly reduced to 15% of the wild type activity when the short chain biguanide metformin was used and increased to 51% for buformin and 76% for phenformin (Figs. 5, *B* and *E*, S8), suggesting that also in these groups of substrates, increasing lipophilicity through longer side chains and the introduction of aromatic features reduced the need for W217 interaction. This correlation between increased molecular weight and lipophilicity with enhanced transport by W217A was also observed in triptans, albeit less pronounced (Fig. 5, *C* and *E*, S8). Interestingly, the correlation between molecular size or lipophilicity and the effect of the W217A mutant was absent in β2-adrenergics (Figs. 5, *D* and *E*, S8).

### Overlapping substrate-specific effects of W217 and F244

**Figure 6:**
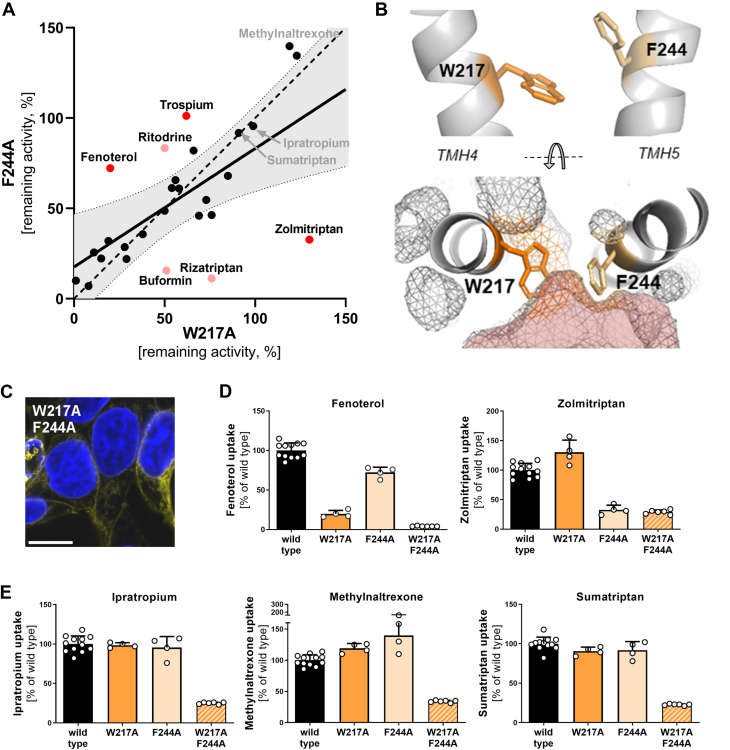
**Role of W217 and F244 for OCT1 polyspecificity**. *A*, comparison of the effects of alanine mutations at codon 217 or 244 on substrate uptake; substrates with significant differences between W217A and F244A are shown in *red*; substrates with substantial differences but no statistical significance are shown in *light red*; statistical significance was determined by ANOVA following Sidak's multiple comparison test corrected for multiple testing using the Holm-Sidak method, with α = 0.05; correlation is represented as regression (*bold* line), 99% confidence interval (*gray* area), and prefect correlation (*dashed* line); shown are means of n = 3 to 4 independent experiments. *B*, localization of W217 and F244 within the protein in outward-occluded state; PDB: 8JTT (27); OCT1 is shown from the side, with the viewpoint centered in the substrate-binding pocket facing TMH4 and TMH5, and the top of the protein oriented toward the extracellular space (*upper* panel) and viewed from the extracellular side (*lower* panel). The OCT1-binding pocket is indicated as *red* mesh. *C*, verification of membrane localization of the W217A F244A double mutant in transiently transfected HEK293 cells; scale bar represents 10 μm; uptake of substrates affected by mutating either W217 or F244 (*D*) or substrates not affected by single mutation (*E*) into transiently (double mutant) or stably transfected (single mutants) HEK293 cells; active uptake was normalized to wild type activity after subtraction of passive diffusion into empty vector control cells; concentrations used are listed in Table S1; shown are means ± SD of n = 4 to 6 independent experiments. OCT1, organic cation transporter 1.

Both W217A and F244A mutants showed substrate-specific effects (Fig. 1). The effects correlated significantly among the 27 substrates tested (Fig. 6*A*, R^2^ = 0.415 and P = 3 × 10^-4^). Thereof, 17 substrates were similarly impacted (within 99% CI) by both mutants, suggesting similar roles of both amino acids. Among them, the transport of small substrates like TEA^+^, metformin, and serotonin (molecular weight < 200 Da, van der Waals volume < 200 Å^3^) was strongly affected by both W217A and F244A mutants (Fig. S9).

Compared to the F244A mutant, the W217A mutant resulted in a stronger decrease in the uptake of fenoterol (3.6-fold), trospium (1.6-fold), and ritodrine (1.7-fold, Fig. 6*A*). In contrast, the F244A but not the W217A mutant showed a substantial reduction in the uptake of zolmitriptan (4-fold), rizatriptan (6.7-fold), and buformin (3.2-fold, Fig. 6*A*), suggesting that one of the two aromatic amino acids is sufficient for the transport.

Structurally, both residues are localized in close proximity (6.4 Å between aromatic rings, 10.3 Å between the Cα backbone atoms), flanking the N-terminal border of the binding pocket (Fig. 6*B*), which is kept throughout the whole transition from outward-open to inward-open state (Fig. S10).

The OCT1 double mutant, which carried both W217A and F244A mutants, was correctly expressed in the plasma membrane (Fig. 6*C*). The double mutant strongly decreased the transport of all substrates tested (Fig. 6, *D* and *E*). Importantly, the uptake of substrates, which were not affected by any of the single mutations, was also reduced to below 40% of the wild type (Fig. 6*E*).

As selected substrates revealed substantial differences in uptake between W217A and F244A, we also analyzed the potential consequences of interactions with inhibitors. Therefore, the opioids morphine and dextromethorphan that were described before to inhibit OCT1-mediated substrate uptake (22) were analyzed for their potency to inhibit ASP^+^ uptake in both mutants (Fig. S11). Regardless of the inhibitor concentration used, no significant differences in ASP^+^ uptake were observed between the mutants and the wild type. Moreover, the inhibition of W217A and F244A was identical.

## Discussion

Despite the availability of cryo-EM structures of OCT1, the overall transport mechanism and especially the molecular basis of its polyspecificity are not fully understood. In this study, we combined functional wet-lab experiments with MD simulations to investigate the roles of W354, W217, and I446—residues that restrict the substrate-binding pocket toward the intracellular lumen. By analyzing the effects of alanine substitutions at these positions on the transport of 27 structurally diverse OCT1 substrates, we observed distinct functional consequences, ranging from complete loss of function for W354 to substrate-dependent effects for W217 and no measurable effect for I446. Notably, we identified divergent roles for the two tryptophans, W354 and W217, despite their similar spatial localization within the protein.

The role of W354 in substrate translocation has been previously discussed (26, 27, 29, 34). Our study expands upon existing experimental data by demonstrating that the functional impact of the W354 mutant extends to a much broader range of substrates, resulting in consistently strong effects across all tested compounds (Figs. 1 and 2). These findings strongly suggest that similar to the YER motif, W354 plays a general and critical role in the conformational switch of the transporter rather than directly interacting with individual substrates. This is in line with Zhang *et al*. (27) who suggested a mechanistic role for W354 and multiple homology models that did not predict any direct interaction of W354 with the substrate (30, 31, 32).

Based on our *in vitro* data, we cannot experimentally distinguish between altered turnover and reduced substrate affinity as causes for the complete loss of function in the W354A mutant. However, several lines of evidence suggest that W354 affects transport rates rather than substrate affinity. First, if W354A mainly disrupted substrate binding, one would expect substrate-specific effects, as seen for W217A, rather than a complete loss of function. Second, cryo-EM structures resolved W354 in the substrate-binding pocket but indicate only weak hydrophobic interactions in the outward-open conformation, making a strong affinity effect unlikely. Third, MD simulations without substrates still showed pronounced effects of W354A on transporter mechanics, consistent with the experimentally observed loss of function.

The fundamental involvement of W354 in OCT1 transport aligns with its high conservation across SLC22 transporters (Fig. S12). Given the diverse substrate selectivities within the SLC22 family, this conservation again suggests that W354 confers a mechanistic role rather than direct substrate interaction, particularly not contributing to polyspecificity. This is in line with cryo-EM structures of other SLC22 transporters, which did not suggest any direct interaction with a substrate or inhibitor (35, 36, 37, 38, 39, 40, 41, 42) while supporting a strong (35) or complete (36) loss of transport activity upon mutation.

In contrast to the YER motif, which is primarily involved in the early conformational transitions of the rocker-switch mechanism—from the outward-open to outward-occluded and inward-occluded states (24, 27)—W354 appears to play an essential role in the transition from the inward-occluded to the inward-open state, facilitating the opening of the binding pocket toward the cytoplasm. Multiple lines of MD-based evidence support this conclusion. First, the most pronounced changes in W354 orientation within the substrate-binding pocket occur during the inward-occluded to inward-open transition (Fig. 3*A*). Second, MD simulations of the W354A and W354F mutants reveal the most substantial deviations from wild type during the inward-open state (Fig. 2, *C*–*E*, Fig. S5). Finally, the interaction between W354 and N453 is disrupted, specifically between the inward-occluded and inward-open states (Fig. 3*A*).

Both our MD and wet-lab data confirm the previously proposed W354–N453 interaction (27). This interaction stabilizes the outward-open state and is disrupted during the final transition to the inward-open conformation (Fig. 3). Whether this interaction dissociates first to trigger pocket opening or is merely a consequence of it remains to be clarified by more detailed MD simulations. However, the strong effects of W354 mutants across all substrates suggest that the W354–N453 interaction acts as a trigger for the conformational switch.

Notably, the YER motif and W354 are structurally linked: both Y361 (of the YER motif) and W354 are located within TMH7, one of the key transmembrane helices involved in the conformational switch. Furthermore, a functional linkage between Y361 and W354 is underscored by the instability of the W354A mutant in inward-open simulations, resulting in a backswitch to an outward-open-like conformation (Fig. 2*E*).

The W354–N453 interaction may be functionally coupled with the YER motif, which connects TMH7, TMH10, and TMH11. In this context, substrate binding may trigger a Y361-driven downward movement of TMH7, associated with the formation of the E386-R439 salt bridge that results after the rotation of TMH10. This concerted motion of TMH7 and TMH10 likely disrupts the hydrogen bond between W354 and N453, leading to the spatial reorientation of W354 (Fig. 3). The resulting loss of hydrophobic contacts with TMHs 10 and 11 facilitates the separation of TMHs 7, 10, and 11, opening the binding pocket toward the intracellular space. Thus, W354 may function as a conformationally responsive anchor within TMH7, stabilizing the outward-facing conformation under resting conditions but also permitting the transition to the inward-open state upon disruption of the W354–N453 interaction. Furthermore, this indicates that the YER motif not only mediates substrate pocket closure (24) but also contributes to its subsequent opening toward the intracellular space.

A common genetic variant in Europeans—G465R—causes a complete loss of transport due to aberrant localization of the protein, probably due to misfolding (10, 43). The W354–N453 interaction might be compromised in the G465R variant (Fig. S13). G465 is located in TMH11 in close spatial proximity to N453. The G465R substitution likely perturbs the local environment around N453 in TMH10, thereby destabilizing the TMH7/10/11 interface. This disruption may lead to protein misfolding and a consequent failure of membrane trafficking.

The substrate-dependent effects of the N453A mutant could derive from a potential partial involvement of N453 in substrate interaction. Given its proximity to the substrate-binding pocket and assuming that the W354–N453 interaction is disrupted during the transition from the inward-occluded to the inward-open conformation, N453 may transiently interact with the substrate as a hydrogen bond donor or acceptor during opening of the substrate-binding pocket toward the intracellular space (Fig. S14). Such interactions could help stabilize the substrate during translocation and facilitate its release into the intracellular space, thereby might indirectly influencing protein turnover. This may be particularly relevant for substrates with high polar surface area and multiple hydrogen bond donors or acceptors, such as fenoterol and methylnaltrexone. In contrast, substrates with minimal hydrogen bonding capacity—such as trospium and ipratropium, each possessing only one hydrogen bond donor—appear to be transported independently of N453. These findings are consistent with previously reported substrate-dependent effects of the N453A mutant on the retained uptake of MPP^+^ but not of metformin (27). However, ipratropium and trospium were transported comparably to the wild type by the double mutant (N453A-C469A). Those substrate-dependent effects could suggest that the transport mechanism is triggered in a substrate-dependent manner. In the case of ipratropium and trospium, conformational changes may be triggered more strongly than sumatriptan and fenoterol, resulting in a stronger movement of TMH7 and 10 and thus still inducing pocket opening even in the absence of N453. However, the specific molecular moieties responsible for this effect remain unclear, particularly since ipratropium and trospium are not transported more efficiently than the other tested substrates under wild type conditions (23). Alternatively, substrate-dependent effects may reflect partial compensation of the missing asparagine in the N453A mutant by substrate structural features, similar to what was observed for Y361A with fenoterol (24).

Despite the similar localization of both tryptophans within the binding pocket, the role of W217 differs substantially from that of W354. W217 appears to be directly involved in substrate interaction. Increasing evidence suggests that it is the aromatic character, rather than hydrogen bonding capability, that is critical for the W217–substrate interaction. Cryo-EM structures of OCT1 consistently show W217 positioned to participate in hydrophobic contacts, cation–π interactions, or π–π stacking with substrates (19, 26, 27). Our mutagenesis data further support the importance of the aromatic ring as, in contrast to W354, the activity of W217 can be partially rescued by substitution with phenylalanine or tyrosine (Fig. 4).

W217 appears to be important for the accommodation of the substrate in the binding pocket rather than for initiating transition. The position of W217 remains stable throughout all transition states, likely due to stabilization *via* a hydrogen bond with S29 (Figs. S10, S15). This suggests that W217’s specific spatial orientation within the binding pocket is crucial for its function. Similar substrate-stabilizing aromatic residues have been identified in organic anion transporters (OATs) and other members of the SLC22 family, forming an aromatic cage that stabilizes diverse substrates through π–π interactions (37).

W217, again in contrast to W354, has strong substrate-specific effects. Substrate size coupled with sufficient lipophilicity appear to be the major determinants of the observed W217 effects. The uptake of small substrates, such as metformin and TEA^+^, was especially dependent on the presence of W217 (Figs. 1 and 5). This may be attributed to the enlarged binding pocket in the W217A mutant, which impairs coordinated interactions with the remaining binding partners by allowing excessive substrate flexibility. This is apparently particularly relevant for small substrates.

The effects of W217 closely correlate with the substrate-dependent effects observed for F244, suggesting that both residues share similar roles in interaction with the substrate (Fig. 6). The proximity of W217 and F244, both restricting the same side of the binding pocket, allows partial compensation in substrate binding when one is absent, particularly for large substrates. However, removing both residues results in a significant loss of transport function (Fig. 6, *D* and *E*). This compensatory mechanism between W217 and F244 was not observed for small substrates (<180 Da), suggesting that these molecules require a coordinated interaction of multiple residues. This is also consistent with observations that metformin uptake was highly sensitive to mutations of individual residues within the substrate-binding pocket (26, 27), whereas MPP^+^ uptake—a more aromatic and therefore, more lipophilic substrate—was primarily affected by mutations in mechanistically critical residues, such as the YER motif, W354, and the K214–D474 salt bridge (27). Consequently, as substrate size increases, the contribution of each individual amino acid (W217 or F244) decreases, with compensation provided by surrounding residues. This is in line with the previously described effects of the F244 mutant on substrate transport, which was associated with small substrates being more strongly affected (20). Differences in interactions involving W217 and F244 likely arise from substrate-dependent binding, where the spatial orientation of the substrate determines whether interaction with W217, F244, or both is favored.

W217 is conserved across OCTs (OCT1–3) only, but not the rest of the SLC22 family, suggesting a role in determining substrate selectivity for cationic compounds (Fig. S12). In comparison, OATs typically feature polar residues—or alanine—at the corresponding position. This structural distinction likely contributes to the divergent substrate specificities between OCTs and OATs. Supporting this, organic cation transporter novel types—which transport cationic and zwitterionic substrates—possess a conserved tyrosine at the analogous position, likely fulfilling a role similar to that of W217. Therefore, W217 might strongly contribute to the recognition and transport of positively charged substrates. In contrast, F244, which shares a similar substrate spectrum, consistently retains an aromatic residue (either phenylalanine, tryptophan, or tyrosine) across paralogs. Notably, W217 is highly conserved across OCT1 orthologs. However, its functional relevance is more pronounced in human OCT1 than in murine OCT1, particularly for small substrates such as serotonin, TEA^+^, and metformin (Fig. 4*D*).

Generally, human and mouse OCT1 share 77% amino acid identity. Direct comparison of the region surrounding W217 reveals several nonconserved residues, including differences within TMH4 as well as two residues in TMH1 (Fig. S16). Among these, the nonconserved residue F32 in hOCT1 (corresponding to L32 in mouse OCT1) has previously been shown to contribute to species-specific differences in substrate selectivity (23). Observed differences may result either from differences in direct interactions between F32 and L32 with the ligand that as a consequence modify the role of W217 or there may be a direct interaction between W217 and F32 in human, as suggested by some of the cryo-EM structures published (26), that is not prone between L32 and W218 in mouse OCT1.

While I446 plays a role in closing the binding pocket from below in the outward-open conformation, its function may be compensated by nearby amino acids such as I449 or C450, which are located in the same TMH10 and also face the binding pocket. Furthermore, the I446A mutant could create additional space for substrates to enter the binding pocket. The importance of position 446 for substrate recognition was previously suggested (26); however, these analyses were performed using the consensus sequence, where phenylalanine occupies this position. Notably, in most OCT1 paralogs, an aromatic amino acid (phenylalanine or tyrosine) is present at this position that can engage direct substrate interactions. (Fig. S12). Thereby, the role of I446 might differ between OCT1 and other SLC22 transporters, being a unique feature for human OCT1.

In conclusion, our data highlight distinct roles for W354 and W217 in OCT1 function and polyspecificity. W354 plays a central mechanistic role by stabilizing the outward-facing conformation and enabling the switch to the inward-open state by the disruption of its interaction with N453. In contrast, W217 contributes to substrate recognition in a substrate-dependent manner. Its role is especially critical for the uptake of small, hydrophilic substrates, while larger or more complex molecules can compensate for its absence through alternative binding sides. The interplay between W217 and F244 further supports a model in which multiple residues collaboratively shape substrate specificity within a flexible binding pocket. Together, these findings refine our understanding of OCT1’s structural determinants of polyspecificity and highlight the necessity of integrating structural data with functional validation to unravel transporter mechanisms.

## Experimental procedures

### Reagents

The chemicals used in this study were sourced as follows: ASP+ was obtained from Life Technologies. Ipratropium bromide, ritodrine hydrochloride, serotonin-d4, sumatriptan-d6, thiamine-d3 hydrochloride, and trospium chloride were purchased from Santa Cruz Biotechnology. Ranitidine-d6 and trospium-d8 were supplied by Toronto Research Chemicals, while buformin hydrochloride was obtained from Wako Chemicals. Additional chemicals, including amisulpride, amisulpride-d5, dobutamine, fenoterol hydrobromide, fenoterol-d6, frovatriptan succinate, metformin hydrochloride, methylnaltrexone bromide, naratriptan hydrochloride, norfentanyl, orciprenaline hemisulfate, phenformin, pirbuterol acetate, ractopamine, rizatriptan benzoate, salbutamol hydrochloride, serotonin, sumatriptan succinate, terbutaline, tetraalkylammonium compounds, thiamine, trimethoprim, and zolmitriptan, were obtained from Sigma-Aldrich. All chemicals were of commercial origin and had a purity of at least 97%.

Dulbecco's modified Eagle's medium (DMEM), Hanks' buffered salt solution (HBSS), fetal bovine serum (FBS), and the Pierce BCA Protein Assay Kit were obtained from Thermo Fisher Scientific. Penicillin-Streptomycin was purchased from PAN-Biotech, while Poly-D-lysine hydrobromide was obtained from Sigma-Aldrich. Hepes was purchased from Carl Roth. Liquid chromatography coupled to tandem mass spectrometer (LC-MS/MS)-grade acetonitrile, methanol, and formic acid were obtained from Merck. Twelve- and twenty-four-well plates were purchased from Starlab, and tissue culture flasks were obtained from Sarstedt.

### Cell lines and cell culturing

The cell lines used in this study included T-REx-293 cells (Life Technologies) and their stably overexpressing variants. Cell lines stably overexpressing the OCT1 alanine mutants W217A, F244A, W354A, or I446A were generated by targeted chromosomal integration using the Flp-In System (Life Technologies) as described before (43, 44, 45). Generated cell lines were validated on DNA and RNA levels to verify the genomic integration and expression. Generation and validation of cell lines expressing the Y361A, E386A, or R439A mutants were described previously (24).

Cells were grown in DMEM supplemented with 10% FBS, 100 U/ml penicillin, and 100 μg/ml streptomycin. Cells were maintained at 37 °C in a humidified environment with 5% CO_2_ and were passaged twice weekly.

### Generation of OCT1 expression constructs

Expression constructs encoding human wild type OCT1 additionally encoded the fluorescent protein mVenus, which was fused to the C terminus of the OCT1 coding sequence. The usage of a fluorescently tagged protein later allowed the direct verification of membrane localization in transfected cells. Generation of the construct, as well as verification of no influence of the tag on OCT1 function, was described previously (24). This expression construct encoding for wild type OCT1 was used to introduce point mutations by site-directed mutagenesis and primers, as listed in Table S2. The integrity of OCT1 expression constructs was verified by enzymatic restriction digest, and mutation sites were verified by capillary sequencing.

### Transient transfection of T-REx-293 cells for cellular uptake experiments

For transient transfection, 5 × 10^5^ T-REx-293 cells were seeded per well in 12-well plates precoated with poly-D-lysine. Twenty-four hours later, cells were transfected. Therefore, cells were washed twice with DMEM containing 10% FBS. Then, 2 μg plasmid diluted in pure DMEM supplemented with 3% Lipofectamine 2000 (Thermo Fisher Scientific) was added per well. After 6 hours, the medium was changed to culture medium containing both 10% FBS and 1% penicillin/streptomycin. At 48 h after transfection, efficacy was assessed by fluorescence microscopy, and cellular uptake experiments were conducted subsequently.

### Cellular uptake experiments

Cellular uptake experiments were performed in 12-well plates for transiently transfected cell lines, whereas 24-well plates were used for stably transfected cell lines. Forty-eight hours before uptake experiments, 3 × 10^5^ cells were seeded per well in 24-well plates precoated with poly-D-lysine. All uptake experiments were performed at single concentrations in technical duplicates. Uptake experiments were conducted at 37 °C, pH 7.4, using HBSS supplemented with 10 mM Hepes (HBSS+). Substrates and concentrations used are listed in Table S1. First, cells were rinsed with prewarmed HBSS+ (0.5 ml for 24-well plates or 1 ml for 12-well plates), and uptake was initiated by adding prewarmed substrates diluted in HBSS+ (180 μl or 400 μl). After 2 min, uptake was stopped by adding ice-cold HBSS+ (400 μl or 2 ml), followed by two washing steps with ice-cold HBSS+ (1 ml or 2 ml). Depending on the later usage of the sample, cells were either lysed using 80% acetonitrile with an internal standard for LC-MS/MS detection or using radioimmunoprecipitation assay buffer for the determination of total protein content and fluorescence spectroscopy. Intracellular substrate concentrations were normalized to the total protein, determined by the bicinchoninic acid assay (46).

### Cellular inhibition experiments

For cellular inhibition experiments, stably transfected cells were seeded in 12-well plates as described above. The experiments followed the same protocol as the uptake assays, with the modification that, in addition to the substrate, an inhibitor was added to the cells. Specifically, cells were exposed to 0.5 μM ASP^+^ in the presence of increasing concentrations of morphine (10 μM, 100 μM, 1000 μM) or dextromethorphan (100 μM, 1000 μM) at their final concentrations. After a 2-min incubation, uptake was stopped by adding ice-cold HBSS+. After two washing steps, cells were lysed in radioimmunoprecipitation assay buffer, and intracellular ASP^+^ concentrations were quantified using fluorescence spectroscopy. Substrate levels were normalized to total protein content, determined *via* the bicinchoninic acid assay (46). All inhibition experiments were performed in technical duplicates.

### Quantification of intracellular substrate concentration

Intracellular substrate concentration was either quantified by LC-MS/MS or for fluorescent substrates such as ASP^+^ by fluorescence spectroscopy.

For LC-MS/MS quantification of intracellular substrate concentrations, cell lysates were centrifuged at 16,000×*g* for 15 min, and 200 μl of the supernatant was evaporated under nitrogen at 40 °C. The residue was reconstituted in 100 μl of 0.1% formic acid, diluted, and 3 to 5 μl was injected into the LC-MS/MS system.

LC-MS/MS analysis was performed using an API6500 QTRAP tandem mass spectrometer with an ESI interface (AB SCIEX) coupled to a Shimadzu LC-40 UHPLC system (Shimadzu). All analytes were detected in positive mode. Chromatographic separation was performed on a Brownlee SPP RP-Amide column (4.6 × 100 mm, 2.7 μm, PerkinElmer) with varying concentrations of organic solvent composed of 90% methanol/ACN (6 + 1) and 0.1% formic acid (solvent A) and pure 0.1% (v/v) formic acid (solvent B, Table S3).

For fluorescence spectroscopy measurement of intracellular ASP^+^ concentrations, cell lysate was directly transferred into black 96-well plates and absorbance was measured in duplicates with an excitation wavelength of 485 nm and emission was detected at 612 nm using the Tecan infinite M200 Microplate Reader (Tecan Group Ltd).

### Confocal microscopy analysis of OCT1-overexpressing cells

To verify that the introduced mutation still allows the protein to be correctly expressed on the membrane, membrane expression was analyzed by confocal microscopy. Therefore, cells were seeded on precoated cover slips in 12-well plates and transiently transfected as described before. For stably transfected cell lines, 6 × 10^5^ cells were seeded. After 48 h, cells were washed three times with Dulbecco's phosphate-buffered saline and then fixed with ice-cold ethanol (100%) at −20 °C for 20 min. Again, cells were washed three times with PBS and coverslips were mounted onto microscope slides using ROTI Mount FluorCare DAPI (Carl Roth). Imaging was performed using a Zeiss LSM780 laser scanning microscope with ZEN 2010 software v6.0. Images were acquired with a 64 × objective, and specific areas were enlarged using the crop function. Contrast and brightness were adjusted using the Fiji software (47). No nonlinear modifications were applied.

### MD simulations

All MD simulations were performed using NAMD 2.14 (48). The initial structure was based on PDB entry 8JTZ (27), representing the outward-facing partially occluded state, which was chosen since it contains no missing residues within the structure. The initial protein preparation was conducted using the Protein Preparation Wizard (Schrödinger Release 2024-2) (49), including optimization of hydrogen-bond assignments using PROPKA at pH 7.4, followed by constrained minimization with a maximum RMSD of 0.3 Å, utilizing the OPLS4 force field (50).

System setup and force field assignments were carried out using CHARMM-GUI (51), applying the CHARMM36 m force field (52) with hydrogen mass repartitioning (53). Posttranslational modifications were incorporated, specifically phosphorylation of residues S333 and T541, as well as glycosylation (GlcNAc) at N71. The membrane positioning was based on data from the OPM database (entry 8ET7) (26), using the human plasma membrane model as detailed in Table S4 (54). The system was solvated with TIP3P water, and ions equaling 150 mM NaCl were added.

For initial equilibration, a multistage protocol was employed: (1) energy minimization for 25,000 steps, (2) 50 ps of *NVT* ensemble dynamics with the protein and membrane retained fixed, (3) an additional 50 ps of *NVT* ensemble with only the protein fixed, using a Tcl forces script to push water molecules from the membrane interior, (4) 200 ps of *NPT* dynamics with fixed protein atoms, and (5) 20 ns of *NPT* simulation with RMSD constraints on the protein backbone, implemented *via* the Colvars module (55, 56).

Following equilibration, a 200 ns production MD simulation was performed with RMSD constraints applied to heavy backbone atoms only. A 4 fs timestep was used, with pressure and temperature controlled *via* a Langevin barostat and Langevin thermostat, respectively. Short-range electrostatic interactions were handled with a cutoff of 1 nm and a switching function starting at 0.9 nm, while long-range electrostatics were treated using the particle mesh ewald method.

The final equilibrated state was employed as the starting point for subsequent targeted molecular dynamics simulations, which modeled transitions between cryo-EM–resolved conformational states along the transport cycle. Structural alignments between states were performed using pairwise alignment from the RCSB database *via* the jCE algorithm (57). To identify stable regions suitable for use as anchor points during targeted molecular dynamics simulations, root mean square fluctuation calculations were performed, highlighting residues 186 to 208 (TMH3, TMH4) and 261 to 269 (TMH6) as relatively stationary throughout the transport process.

Targeted molecular dynamics simulations were conducted to transition between these states, employing an RMSD collective variable with a force constant of 1440 kcal/(mol·Å^2^), using the Colvars module. After reaching each target state, two independent 200 ns NPT simulations were performed: one with backbone constraints and one without, to ensure adequate structural relaxation.

The final snapshot from each constrained simulation was used for subsequent mutation analysis, specifically the W354A and W354F mutants. These were introduced using psfgen 2.0, followed by short energy minimization and subsequent 200 ns *NPT* simulations without restrains using a 2 fs timestep. All other parameters were kept identical to those described above.

Throughout all simulations, trajectory snapshots were recorded every 10 ps. The analysis of all simulations was conducted using in-house Python scripts based on the MDAnalysis 2.8.0 library (58).

### Data analyses

Percentual uptake of mutants relative to the wild type was calculated as uptake (mutant)/uptake (wild type) after subtraction of passive diffusion obtained from empty vector controls. All uptake studies of substrates at single concentration were performed in duplicates and means were represented in graphs. When the data is presented as a percentage of the wild type, the variability of the wild type samples represents the variability of the two biological replicates performed in each experiment. ASP^+^ uptake inhibition was assessed relative to the noninhibited control for each cell line. To account for passive diffusion, values from empty vector controls were subtracted. Inhibition was then normalized to the ASP^+^ uptake in the respective cell line incubated solely with ASP^+^ in the absence of inhibitors.

Correlation analysis of mutant effects on the uptake of multiple substrates was conducted using a two-tailed Pearson correlation test. Differences in uptake between mutants for individual substrates were assessed using ANOVA, followed by Sidak's multiple comparison test, with correction for multiple testing using the Holm–Sidak method. All statistical analyses and data visualizations were performed using GraphPad Prism version 8.01 (GraphPad Software Inc., La Jolla, CA).

We used PyMOL (The PyMOL Molecular Graphics System, Version 2.5.7, Schrödinger, LLC) for visualization and determination of distances between residues of already published cryo-EM structures. CASTpFold (59) was used to calculate and visualize the substrate-binding pocket in selected MD simulations using a 2 Å cutoff. Ketcher 3.3.0 was used for drawing and displaying of chemical structures whereas Chemicalize was used for characterizing chemical structures (June 2025), https://chemicalize.com/, developed by ChemAxon.

## Data availability

All data described is contained within this manuscript and supporting information.

## Supporting information

This article contains supporting information (15, 20, 22, 23,24 , 27, 54, 60, 61, 62, 63, 64).

## Conflict of interest

The authors declare that they have no conflicts of interest with the contents of this article.
