# Supplementary material for: The divergent roles of tryptophans W354 and W217 in OCT1 transport: Similar localization, distinct functions
Source: J Biol Chem. 2025 Dec 13;302(2):111051. doi: 10.1016/j.jbc.2025.111051 (PMC12853787; doi:10.1016/j.jbc.2025.111051)
Supplement: Supplementary Material 1 [file mmc1.docx]

# Supporting information

**The divergent roles of tryptophans W354 and W217 for OCT1 transport: similar localization, distinct functions**

Sarah Römer^1^, Lennart Ebel², Anna Neumann^1^, Vincent Rönnpagel^1^, Lukas Schulig^2^, Marleen J. Meyer-Tönnies^1^, Mladen V. Tzvetkov^1^*

^1^ Department of General Pharmacology, Institute of Pharmacology, Center of Drug Absorption and Transport (C_DAT), University Medicine Greifswald, Greifswald, Germany

^2^ Department of Pharmaceutical and Medicinal Chemistry, University Greifswald, Greifswald, Germany

**Table of contents**

[**Figure S1. S-2**](#_Toc213915915)

[**Figure S2. S-2**](#_Toc213915916)

[**Figure S3. S-3**](#_Toc213915917)

[**Figure S4. S-4**](#_Toc213915918)

[**Figure S5. S-5**](#_Toc213915919)

[**Figure S6. S-5**](#_Toc213915920)

[**Figure S7. S-6**](#_Toc213915921)

[**Figure S8. S-7**](#_Toc213915922)

[**Figure S9. S-8**](#_Toc213915923)

[**Figure S10. S-9**](#_Toc213915924)

[**Figure S11. S-10**](#_Toc213915925)

[**Figure S12. S-11**](#_Toc213915926)

[**Figure S13. S-12**](#_Toc213915927)

[**Figure S14. S-12**](#_Toc213915928)

[**Figure S15. S-13**](#_Toc213915929)

[**Figure S16. S-14**](#_Toc213915930)

[**Table S1. S-15**](#_Toc199974642)

[**Table S2. S-16**](#_Toc199974643)

[**Table S3. S-17**](#_Toc199974644)

[**Table S4. S-18**](#_Toc199974645)

**References ………………………………………………………………………………………………………………………………………… . S-19**


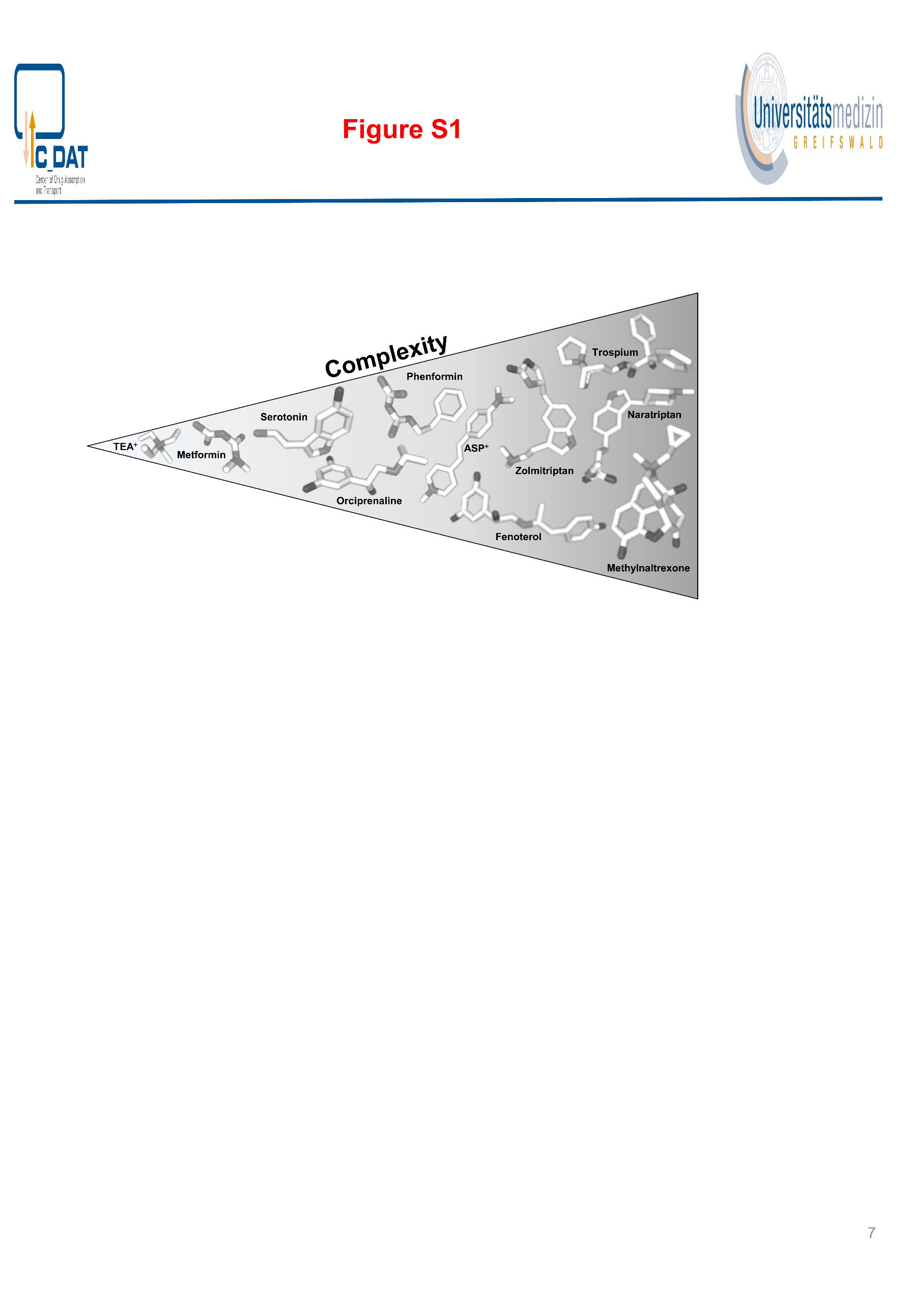


**Figure S1. Structural diversity of OCT1 substrates.** Representative OCT1 substrates are ordered in increasing complexity covering a broad spectrum of structural different compounds. Complexity and 3D structures were derived from pubchem.com (61).


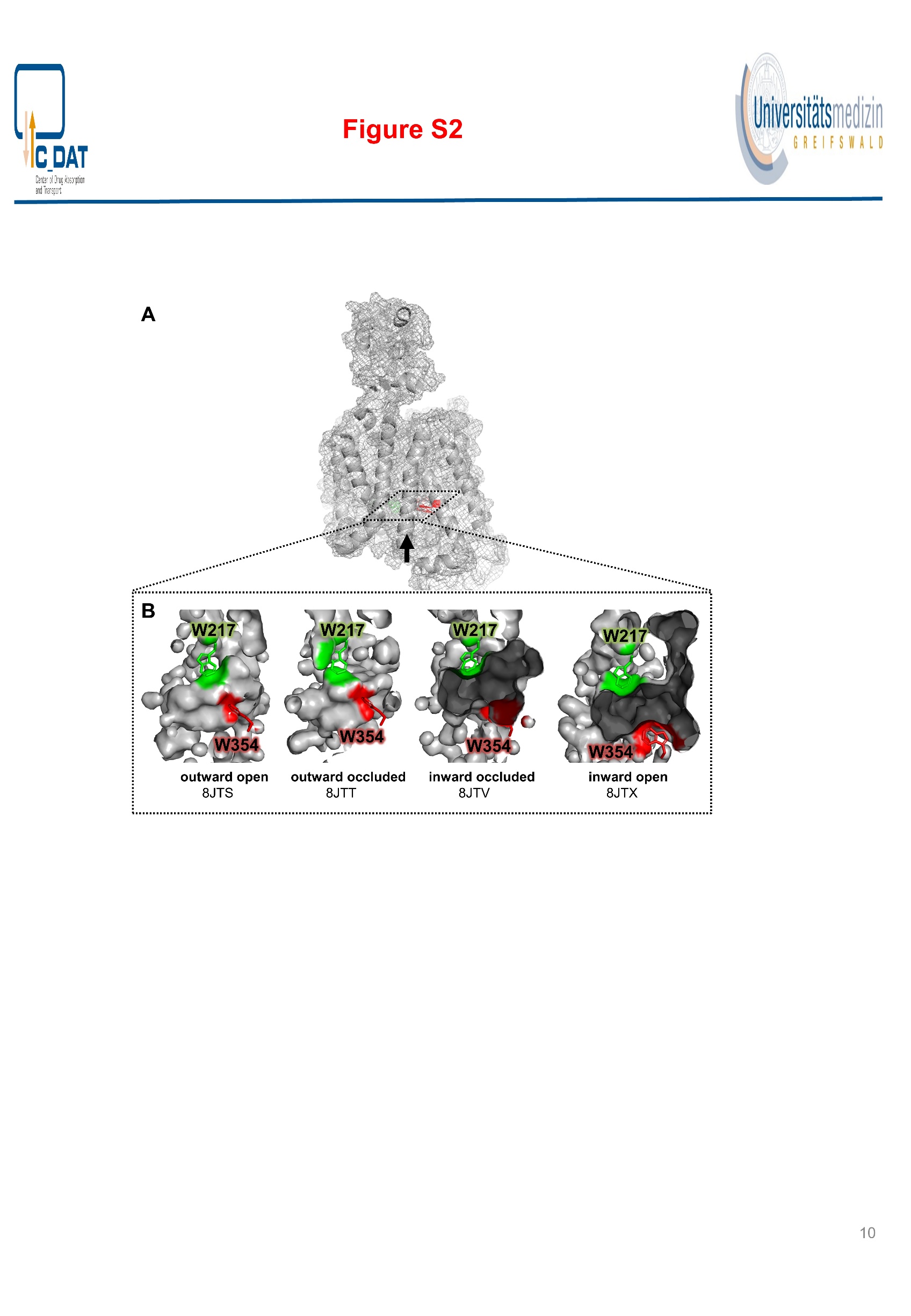


**Figure S2.** **W217 and W354 contribute to the closure and opening of the substrate-binding pocket toward the intracellular lumen.** A) Overview of the protein showing helices and surface in the inward-open state (PDB: 8JTX). B) Opening of the substrate-binding pocket toward the intracellular lumen during transition from outward-open to the inward-open state. OCT1 is shown from the intracellular side viewing the inner gate of the substrate-binding pocket that opens during translocation toward the intracellular lumen. Individual PDB identifiers are listed below the corresponding structure (27).

**Figure S3. Effects of W354 mutation on transport.** Uptake of key OCT1 substrates into HEK293 cells transiently transfected with different mutants of W354; active uptake was normalized to wild type activity after subtraction of passive diffusion into empty vector control cells; concentrations used are listed in Table S1; shown are means ± SD of n=4-6 independent experiments


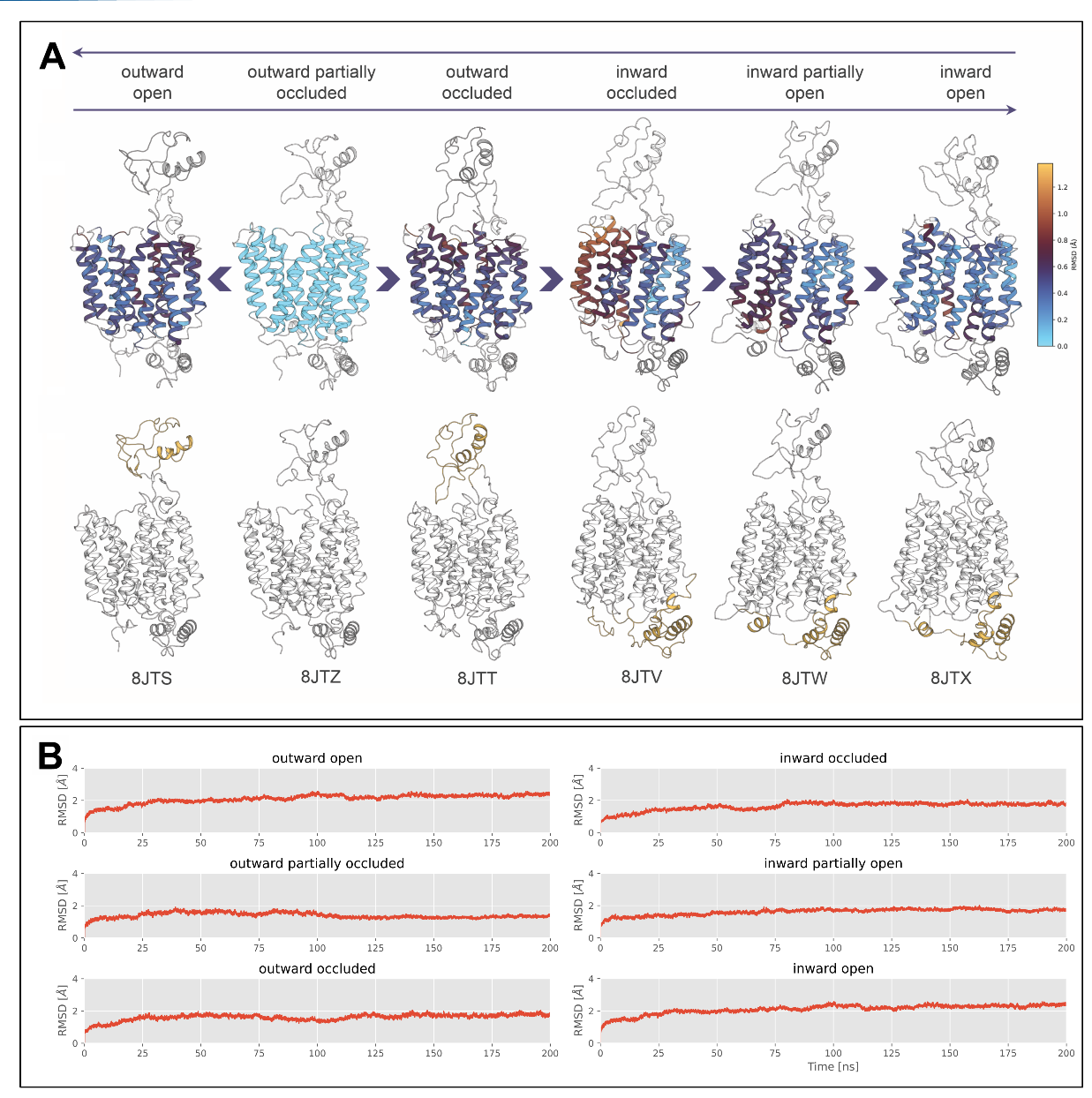


**Figure S4. MD simulations of OCT1 resolving structural changes between each state.** A) Complete protein structures of each conformational state, reconstructed from available cryo-EM data using targeted molecular dynamics simulations (upper panel). The outward-facing partially occluded state was used as the initial structure, since it contains no missing residues within the structure. Structures are colored based on the RMSD of the Cα atoms relative to the preceding state, highlighting regions of conformational change. Missing residues in each original cryo-EM structure are highlighted in gold for their respective PDB entries (lower panel). B) Root-mean-square deviation (RMSD) plots for the transmembrane region of all simulated wild type states after targeted molecular dynamics simulation.


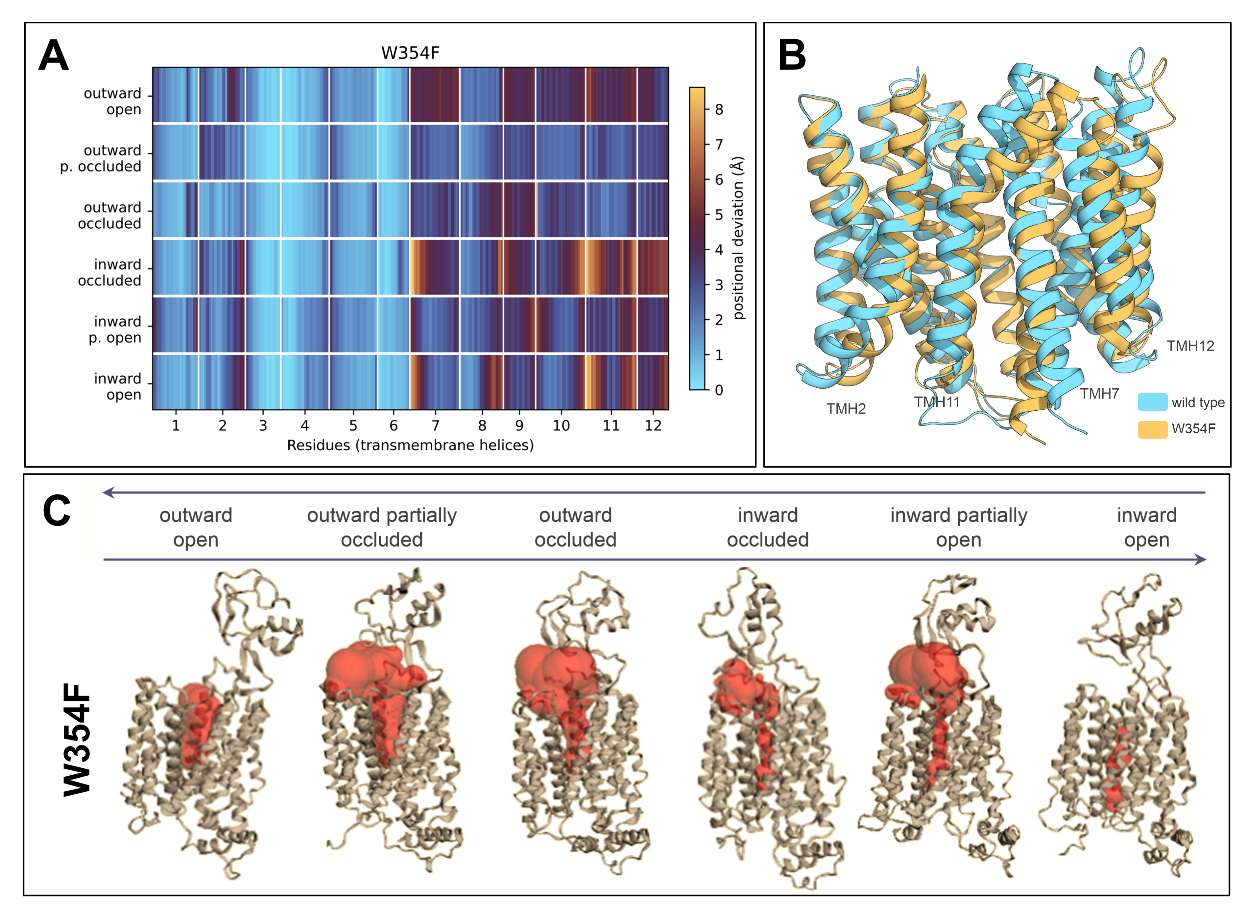


**Figure S5. Stability of the W354F mutant across different conformational states.** A) RMSD of each Cα atom comparing the mutant protein (W354F) to the wild type conformation for each state. Mutant structures were generated analogously to W354A by averaging the last 500 snapshots from three independent molecular dynamics simulations. The W354 mutation also induces clear perturbations of the protein structure and similar structural collapses and rearrangements. B) Structural alignment of the wild type (blue) and W354F mutant (orange) in the inward-open state exemplarily displays substantial conformational changes. C) Effect of W354F mutation on protein conformation and substrate-binding pocket (red) across all conformational states. OCT1 is shown from the side with the top of the protein facing the extracellular space.

**Figure S6. Effects of W217 mutation on transport.** Uptake of key OCT1 substrates into HEK293 cells transiently transfected with different mutants of W217; active uptake was normalized to wild type activity after subtraction of passive diffusion into empty vector control cells; concentrations used are listed in Table S1; shown are means ± SD of n=3-5 independent experiments


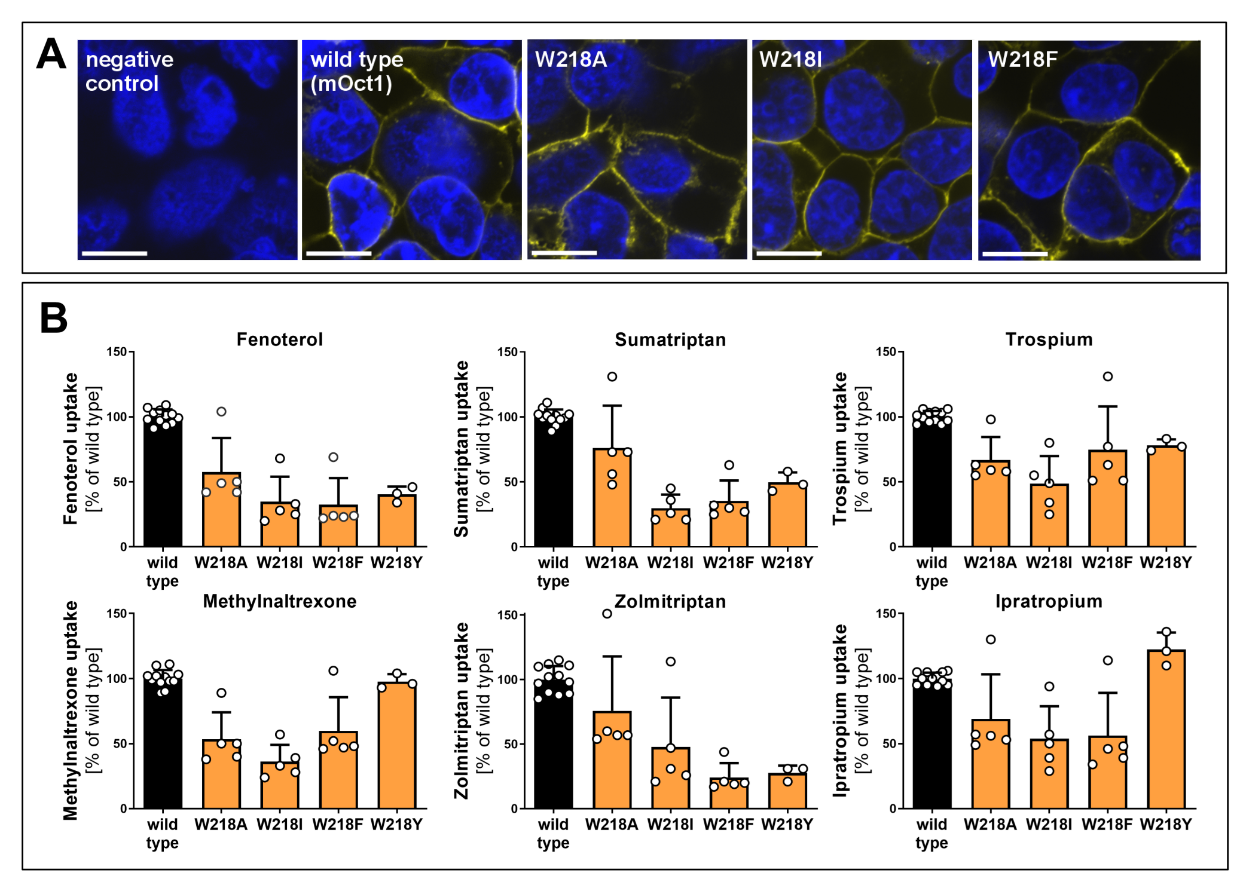


**Figure S7. Effects of W218 mutation in murine OCT1 (mOCT1) on transport.** Uptake of key OCT1 substrates into HEK293 cells transiently transfected with different mutants of W218 in mOCT1, corresponding to W217 in human OCT1; active uptake was normalized to wild type activity after subtraction of passive diffusion into empty vector control cells; concentrations used are listed in Table S1; shown are means ± SD of n=3-5 independent experiments


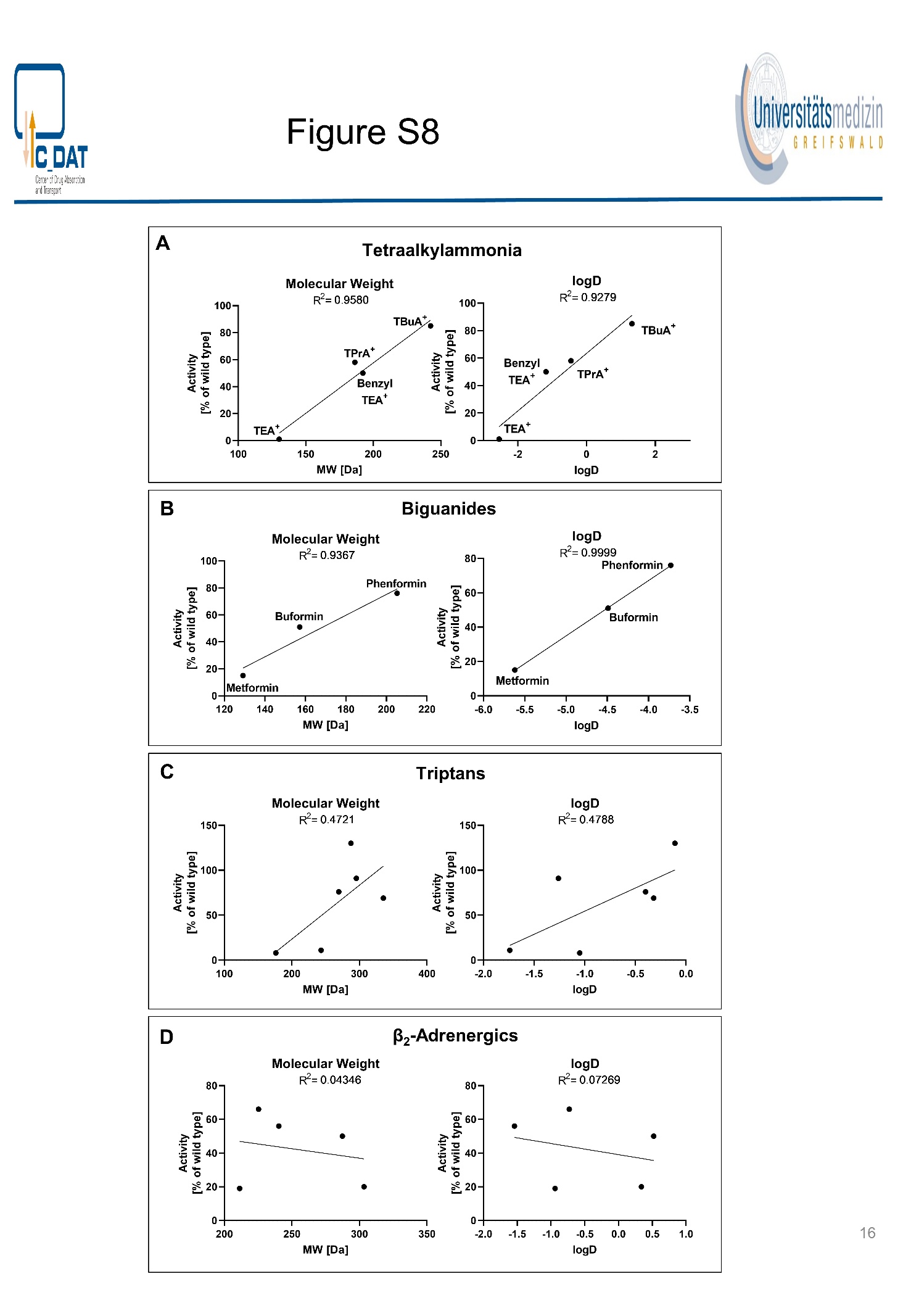


**Figure S8. Correlation between molecular weight and lipophilicity of selected drug classes on uptake by the W217A mutant.** Uptake of key OCT1 substrates into HEK293 cells stably transfected with the W217A mutant; active uptake was normalized to wild type activity after subtraction of passive diffusion into empty vector control cells; concentrations used are listed in Table S1; shown are means of n=3-5 independent experiments

**Figure S9. Molecular characteristics of analyzed substrates and their influence on transport by the W217A or F244A mutant.** Analyzed substrates were divided into affected drugs (<50% wild type activity, black) and not affected drugs (>50% wild type activity, white) depending on the transport activity mediated by the W217A or F244A mutant. Substrates with 50% ± 10% activity were excluded from analysis. Statistical analysis was performed using one-way ANOVA following Tukey’s multiple comparisons test; * p<0.05; ** p<0.01


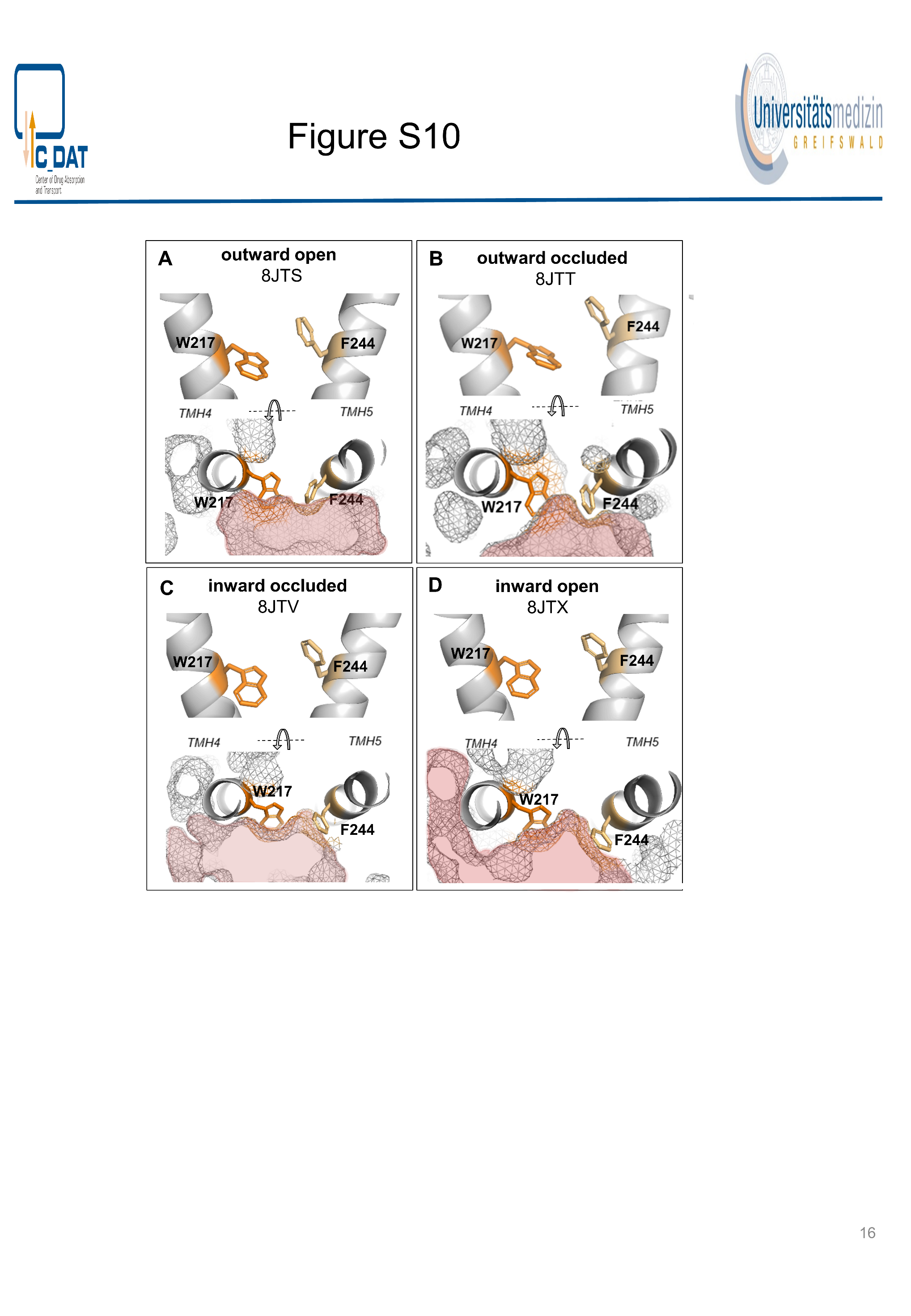


**Figure S10. W217 and F244 share similar localization and movement during all conformational transitions.** Transition from outward occluded (PDB: 8JTT) to inward occluded state (PDB: 8JTV) is followed by slight downward movement of both W217 and F244 maintaining the distances between both residues and flanking the substrate-binding pocket in both conformations (27). OCT1 is shown from the side, with the viewpoint centered in the substrate-binding pocket facing the TMH4 and TMH5, and the top of the protein oriented toward the extracellular space (upper panel) and viewed from the extracellular side (lower panel). The substrate-binding pocket is indicated as red mesh.

**Figure S11. Influence of W217A or F244A mutation on inhibition of ASP^+^ uptake by morphine and dextromethorphan.** Cells stably overexpressing wild-type OCT1 or the W217A or F244A mutants were incubated with 0.5 µM ASP+ and varying concentrations of either morphine or dextromethorphan for 2 min. Uptake of ASP^+^ was normalized on uninhibited uptake of each cell line. Differences in uptake were tested for statistical significances using the Tukey's multiple comparisons test. No statistical difference was observed. Shown are means and range of n=3 independent experiments.


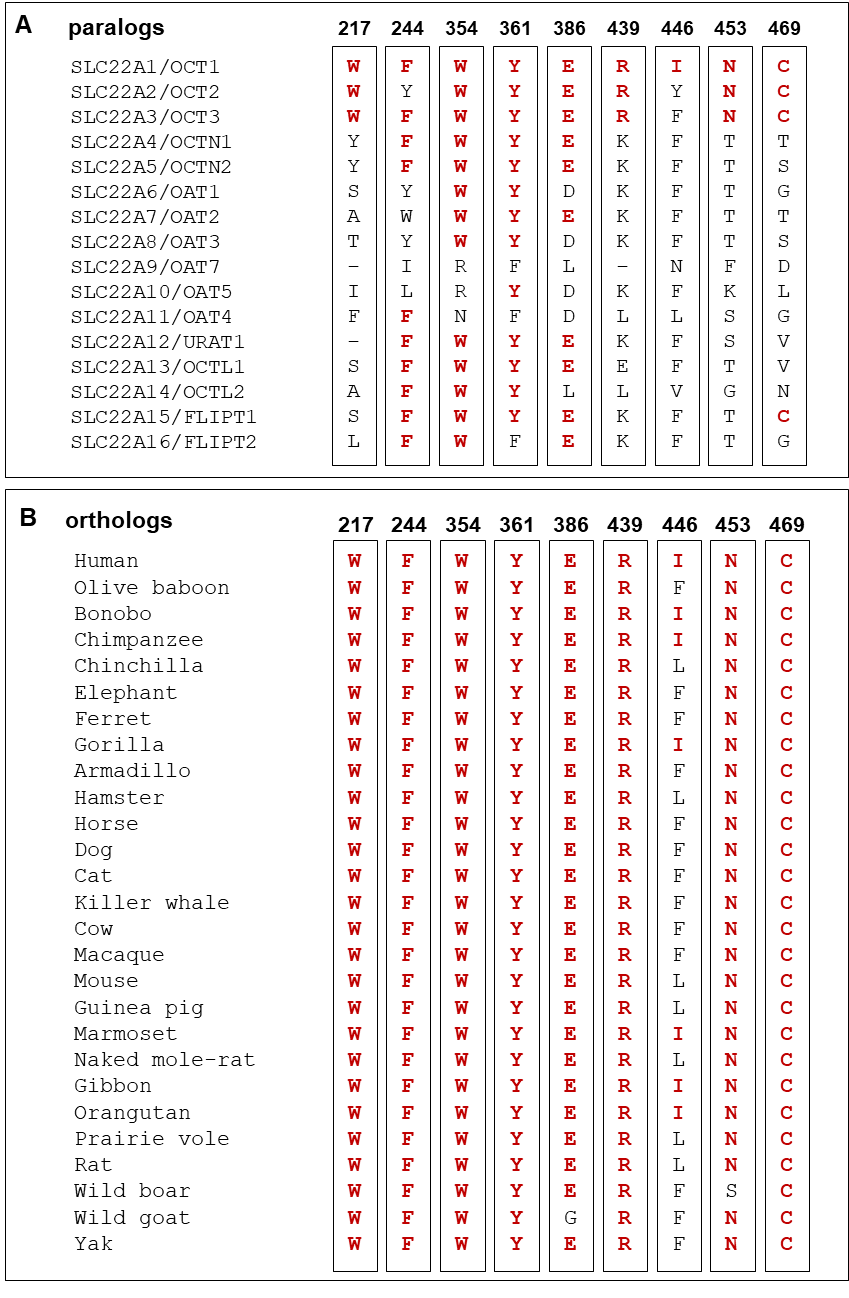


**Figure S12. Alignment of amino acid sequences of SLC22 paralogs (A) and OCT1 orthologs (B).** Similarity to human OCT1 sequence is indicated in red.


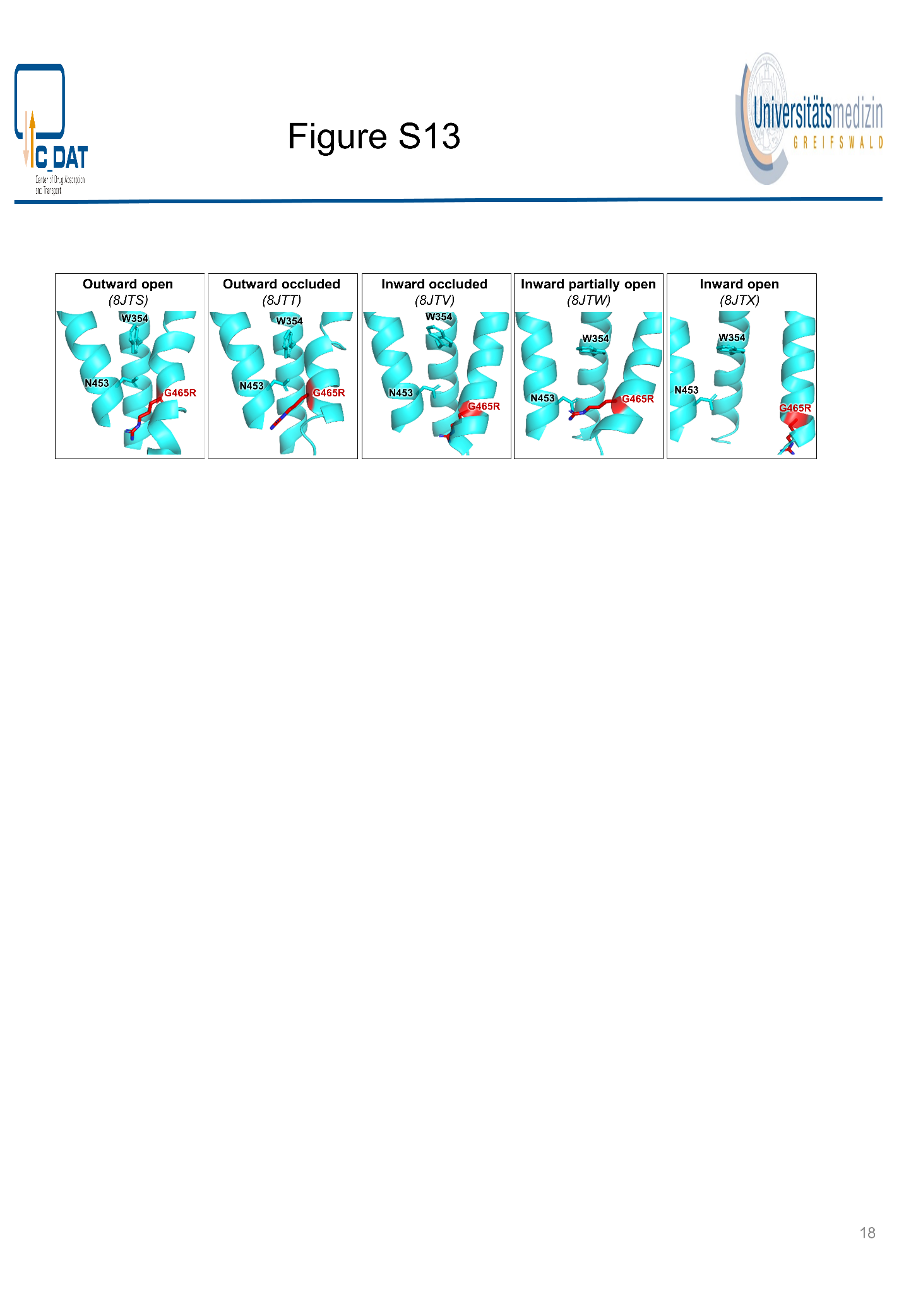


**Figure S13. Local proximity of the G465R variant can compromise the W354-N453 interaction resulting in the clinically relevant loss of function.** The G465R variant (red) was introduced in available cryo-EM structures using PyMOL (Version 2.5.7, Schrödinger, LLC). Individual PDB identifiers are listed below the corresponding structure (27). OCT1 is shown from the side, with the viewpoint centered in the substrate-binding pocket facing the TMH7/10/11 interface, and the top of the protein oriented toward the extracellular space.


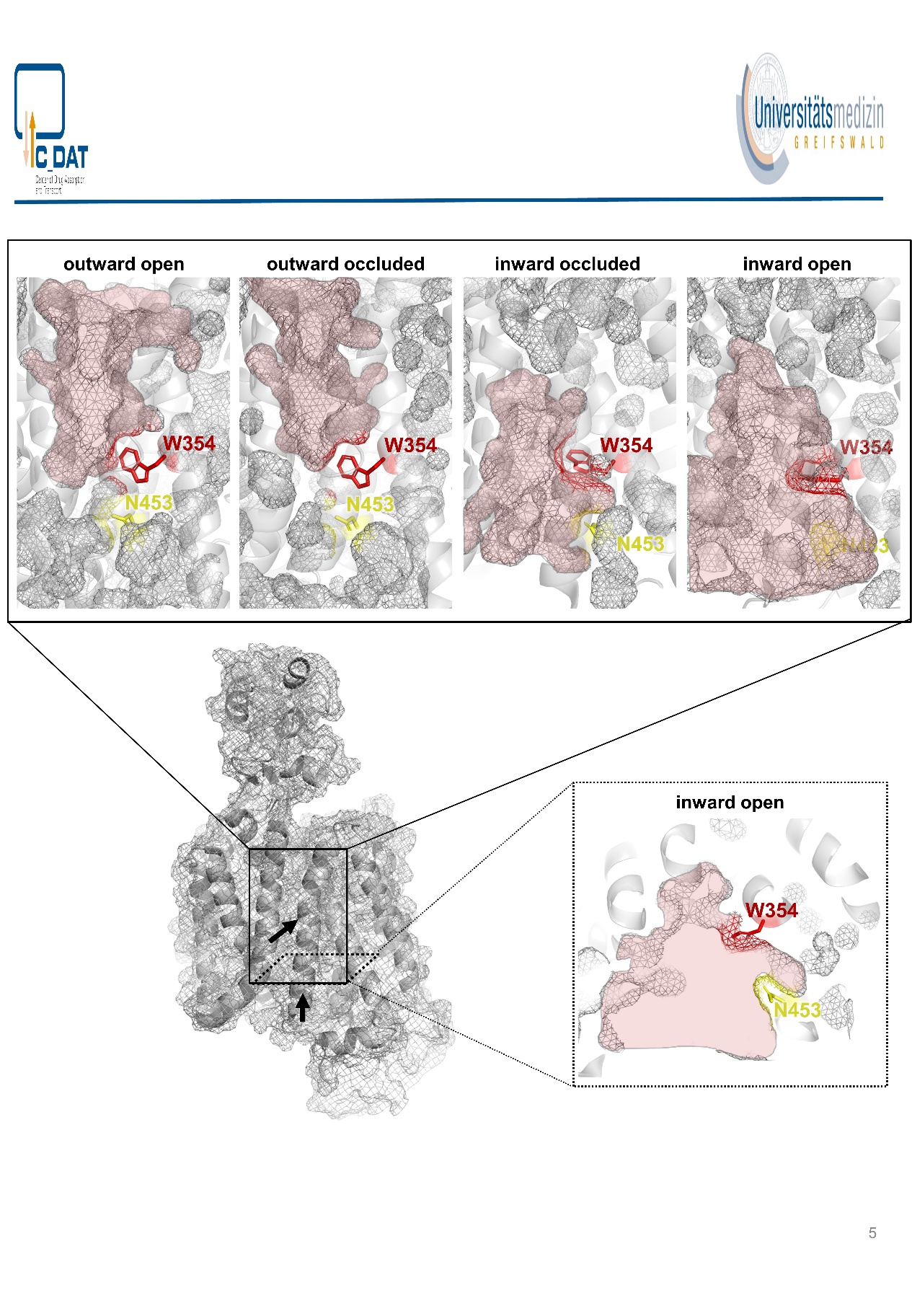


**Figure S14. Localization of W354 and N453 throughout conformational changes.** Complete OCT1 protein is shown in side view, with the top of the protein facing the extracellular space, in the inward-open conformation (PDB: 8JTX). The substrate-binding pocket is highlighted and magnified from different perspectives (PDB: 8JTS, 8JTT, 8JTV, 8JTX) (27). The pocket is viewed either from the side (solid box) or from the intracellular side (dashed box), with viewing directions indicated by arrows. The substrate-binding pocket is depicted as a red mesh, and the overall protein surface is shown in grey mesh.


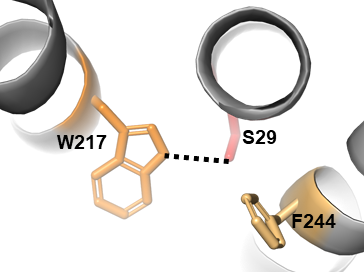


**Figure S15. Potential hydrogen bond between W217 and S29 stabilizes the spatial orientation of W217 of OCT1 in inward-open state (PDB: 8JTX) (27).**


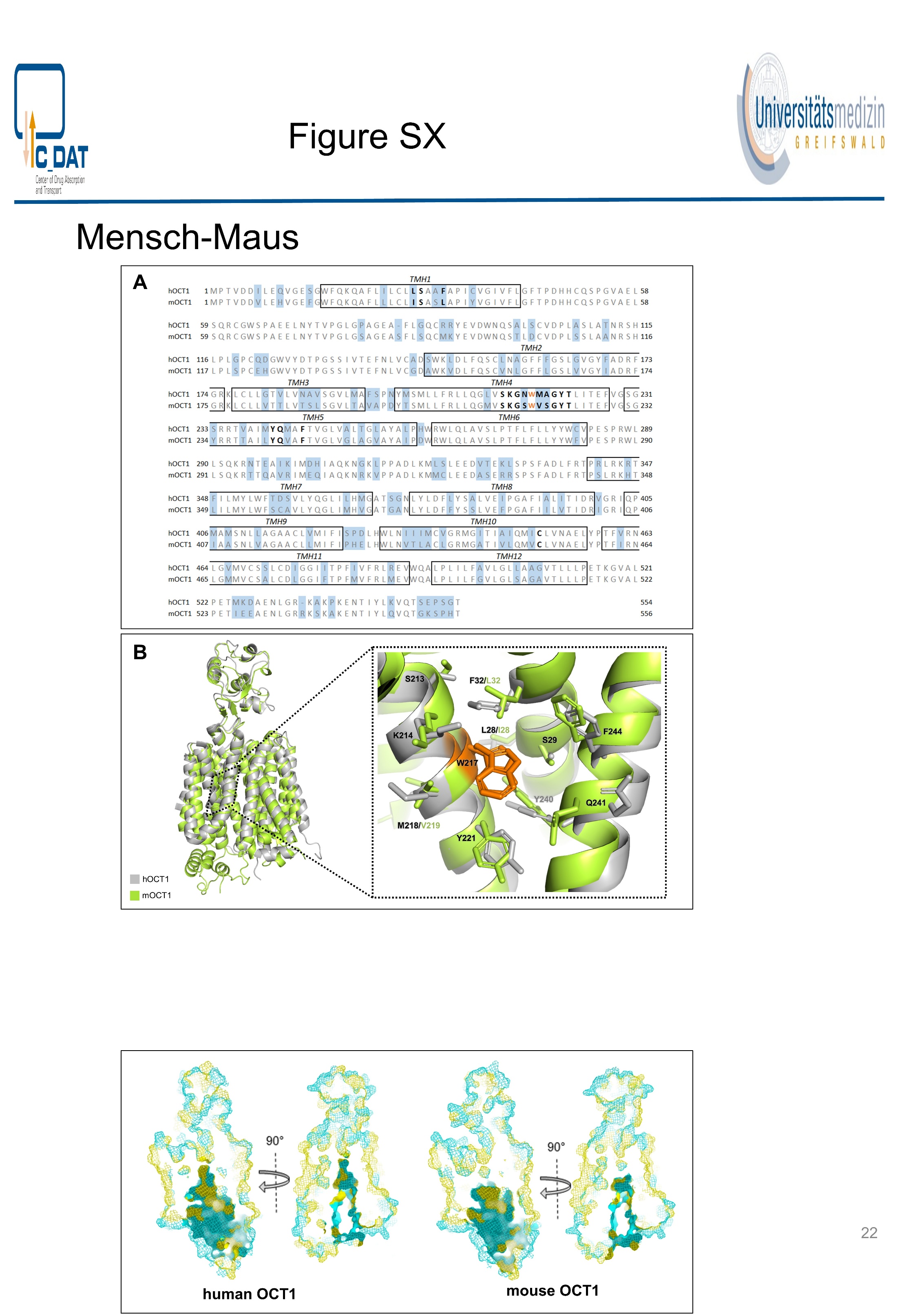


**Figure S16. Structural comparison of human and mouse OCT1.** A) Amino acid sequence alignment of human OCT1 (hOCT1) and mouse OCT1 (mOCT1). Residues located within 5 Å of W217 in hOCT1 (corresponding to W218 in mOCT1) are shown in bold. Non-conserved residues are highlighted in light blue. Transmembrane helices (TMHs) are annotated according to available OCT1 cryo-EM structures. B) Structural comparison of hOCT1 and mOCT1 in the inward-open conformation. The region within 5 Å of W217 (orange) is shown from the substrate-binding pocket toward the membrane plane. Conserved residues are annotated based on hOCT1 numbering, whereas non-conserved residues are labeled for hOCT1 (black) and mOCT1 (green). The mOCT1 structure was predicted using AlphaFold3 (64).

**Table S1. Substrates and concentrations used for uptake experiments.** Concentrations were chosen to be below known K_M_.

| **Substrate class** | **Substrates** | **Concentration used**  **[µM]** | **K_M_** | **Reference for K_M_** |
| --- | --- | --- | --- | --- |
| **Tetraalkylammonium compounds**  **(nTAA)** | Tetraethylammonium (TEA^+^) | 10 | 749 | (23) |
|  | Benzyltriethylammonium (Benzyl-TEA^+^) | 10 | 38.6 | (15) |
|  | Tetrapropylammonium (TPrA^+^) | 10 | 11.4 | (23) |
|  | Tetrabutylammonium (TBuA^+^) | 10 | 26.0 | (24) |
| **Antidiabetics (Biguanides)** | Metformin | 100 | 1470 | (23) |
|  | Buformin | 5 | 149 | Own unpublished data |
|  | Phenformin | 5 | 18.7 | (23) |
| **Triptans** | Sumatriptan | 0.5 | 65.9 | (23) |
|  | Zolmitriptan | 0.5 | 98.5 | (20) |
|  | Frovatriptan | 1 | N/A | N/A |
|  | Naratriptan | 1 | N/A | N/A |
|  | Rizatriptan | 1 | N/A | N/A |
| **Tropane alkaloids** | Ipratropium | 0.5 | 10.7 | (23) |
|  | Trospium | 0.1 | 17.0 | (23) |
| **Beta adrenergic**  **agonists** | Ritodrine | 0.5 | 0.68 | (23) |
|  | Fenoterol | 0.5 | 0.87 | (23) |
|  | Orciprenaline | 200 | 530 | (23) |
|  | Salbutamol | 5 | 395 | (23) |
|  | Pirbuterol | 20 | 29,2 | (23) |
|  | Terbutaline | 100 | 197 | (23) |
| **Endogenous**  **compounds** | Thiamine | 100 | 1057 | (60) |
|  | Serotonin | 1 | 663 | (23) |
| **Opioids** | Methylnaltrexone | 1 | 10.0 | (23) |
|  | Norfentanyl | 0.1 | 7.7 | (22) |
| **Model substrates** | ASP+ | 5 | 63 | (23) |
| **Other** | Amisulpride | 3 | 31 | (62) |
|  | Ranitidine | 1 | 62.9 | (63) |

N/A not available

**Table S2. Primers used for site-directed mutagenesis to introduce point mutations.**

| **Construct** | **Primer name** | **Sequence (5’->3’)** |
| --- | --- | --- |
| **W217A** | W217A_for | CTGGTCAGCAAGGGCAACGCGATGGCTGGCTACACCCTA |
|  | W217A_rev | TTAGGGTGTAGCCAGCCATCGCGTTGCCCTTGCTGACCAG |
| **W217I** | W217I_for | CTGGTCAGCAAGGGCAACATTATGGCTGGCTACACCCTAA |
|  | W217I_rev | TTAGGGTGTAGCCAGCCATAATGTTGCCCTTGCTGACCAG |
| **W217F** | W217F_for | CTGGTCAGCAAGGGCAACTTTATGGCTGGCTACACCCTAA |
|  | W217F_rev | TTAGGGTGTAGCCAGCCATAAAGTTGCCCTTGCTGACCAG |
| **W217Y** | W217Y_for | CTGGTCAGCAAGGGCAACTATATGGCTGGCTACACCCTAA |
|  | W217Y_rev | TTAGGGTGTAGCCAGCCATATAGTTGCCCTTGCTGACCAG |
| **F244A** | F244A_for | ATCATGTACCAGATGGCCGCCACGGTGGGGCTGGTGGCG |
|  | F244A_rev | CGCCACCAGCCCCACCGTGGCGGCCATCTGGTACATGAT |
| **W354A** | W354A_for | TTCATCCTGATGTACCTGGCGTTCACGGACTCTGTGCTC |
|  | W354A_rev | GAGCACAGAGTCCGTGAACGCCAGGTACATCAGGATGAA |
| **W354I** | W354I_for | TTCATCCTGATGTACCTGATTTTCACGGACTCTGTGCTC |
|  | W354I_rev | GAGCACAGAGTCCGTGAAAATCAGGTACATCAGGATGAA |
| **W354L** | W354L_for | TTCATCCTGATGTACCTGCTTTTCACGGACTCTGTGCTC |
|  | W354L_rev | GAGCACAGAGTCCGTGAAAAGCAGGTACATCAGGATGAA |
| **W354F** | W354F_for | TTCATCCTGATGTACCTGTTTTTCACGGACTCTGTGCTC |
|  | W354F_rev | GAGCACAGAGTCCGTGAAAAACAGGTACATCAGGATGAA |
| **W354Y** | W354Y_for | TTCATCCTGATGTACCTGTATTTCACGGACTCTGTGCTC |
|  | W354Y_rev | GAGCACAGAGTCCGTGAAATACAGGTACATCAGGATGAA |
| **I446A** | I446A_for | ATGGGAATCACCATTGCAGCACAAATGATCTGCCTGGTG |
|  | I446A_rev | CACCAGGCAGATCATTTGTGCTGCAATGGTGATTCCCAT |
| **N453A** | N453A_for | CAAATGATCTGCCTGGTGGCTGCTGAGCTGTACCCCACA |
|  | N453A_rev | TGTGGGGTACAGCTCAGCAGCCACCAGGCAGATCATTTG |
| **C469A** | C469A_for | AACCTCGGAGTGATGGTGGCTTCCTCCCTGTGTGACATA |
|  | C469A_rev | TATGTCACACAGGGAGGAAGCCACCATCACTCCGAGGTT |

**Table S3. LC-MS/MS methods for quantifying the intracellular contents of individual compounds after uptake measurements.** Each LC-MS/MS method (I-V) allowed the simultaneous quantification of 3 to 11 compounds (including internal standards)

| **Method** | **Analyte** | **Mass transition (Q1>Q3)** | **Internal Standard** | **Mass transition internal standard (Q1>Q3)** | **Chromatographic separation** | **Flow rate** | **Injection volume** |
| --- | --- | --- | --- | --- | --- | --- | --- |
| I | Metformin | 130.1>71.0 | - | - | 0-5 min: 0-20% A  5-7 min: 20% - 80% A  7-9.5 min: 0% A | 0.55  ml/min | 5 µL |
|  | Sumatriptan | 296.3>57.9 | Sumatriptan-d6 | 302.1>64.1 |  |  |  |
|  | Zolmitriptan | 288.0>58.0 |  |  |  |  |  |
|  | Frovatriptan | 244.4>213.3 |  |  |  |  |  |
|  | Naratriptan | 336.1>98.3 |  |  |  |  |  |
|  | Rizatriptan | 270.0>158.0 |  |  |  |  |  |
|  | Ipratropium | 332.2>124.2 | Trospium-d8 | 400.1>190.1 |  |  |  |
|  | Trospium | 392.1>164.0 |  |  |  |  |  |
|  | Methylnaltrexone | 356.2>338.2 |  |  |  |  |  |
| II | Fenoterol | 304.5>135.0 | Fenoterol-d6 | 310.3>109.1 | 0-4 min: 2% A  4.1-7 min: 15% A  7-9 min: 15%-30% A  9.1-9.6 min: 80% A  9.7-12 min: 2% A | 0.55  ml/min | 5 µL |
|  | Salbutamol | 240.5>222.0 |  |  |  |  |  |
|  | Pirbuterol | 241.0>167.2 |  |  |  |  |  |
|  | Orciprenaline | 212.0>194.3 |  |  |  |  |  |
|  | Ritodrine | 288.0>270.0 |  |  |  |  |  |
|  | Terbutaline | 226.0>152.2 |  |  |  |  |  |
| III | Tetraethylammonium (TEA^+^) | 130.0>86.0 | Tetramethyl-ammonium  (TMA^+^) | 74.2>58.3 | 0-0.5 min: 30%A  0.5-1.5 min: 30-50% A  1.5-3 min: 50-85% A  3-4.1 min: 85% A  4.2-5.5 min: 30 % A | 0.65  ml/min | 3 µL |
|  | Benzyl-Triethylammonium  (Benzyl-TEA^+^) | 192.0>90.9 |  |  |  |  |  |
|  | Tetrapropylammonium (TPrA^+^) | 185.9>114.1 |  |  |  |  |  |
|  | Tetrabutylammonium (TBuA^+^) | 242.1>142.3 |  |  |  |  |  |
| IV | Amisulpride | 370.3>242.0 | Amisulpride-d5 | 375.1>241.9 | 0-2 min: 3% A  2-6 min: 3-20% A  6-8.1 min: 20% A  8.1-9 min: 80% A  9-11 min: 3% A | 0.5  ml/min | 5 µL |
|  | Ranitidine | 314.9>176.2 | Ranitidine-d6 | 321.2>176.0 |  |  |  |
|  | Thiamin | 264.9>122.0 | Thiamin-d3 | 269.1>125.0 |  |  |  |
|  | Serotonin | 177.0>160.0 | Serotonin-d4 | 180.8>163.9 |  |  |  |
|  | Norfentanyl | 232.8>84.2 | Fenoterol-d6 | 310.3>109.1 |  |  |  |
| V | Buformin | 158.1>60.0 | Metformin | 130.1>71.0 | 0-4 min: 5% A | 0.5  ml/min | 5 µL |
|  | Phenformin | 206.5>60.2 |  |  |  |  |  |

**Table S4. Asymmetric mammalian plasma membrane used for molecular dynamics simulations of all systems (54).**

| **Lipid Name** | **Lipid Head/Tail** | **Outer** | **Inner** |
| --- | --- | --- | --- |
| POPC | PC(16:0/18:1(9*Z*)) | 30 | 14 |
| PLPC | PC(16:0/18:2(9*Z*, 12*Z*)) | 42 | 22 |
| PAPE | PE(16:0/20:4(5*Z*,8*Z*,11*Z*,14*Z*)) | 6 | 24 |
| POPE | PE(16:0/18:1(9*Z*)) | 6 | 28 |
| POPI | PI(16:0/18:1(9*Z*)) | 0 | 10 |
| PAPS | PS(16:0/20:4(5*Z*,8*Z*,11Z,14*Z*)) | 0 | 22 |
| POPA | PA(16:0/18:1(9*Z*)) | 0 | 2 |
| SSM | SM(d18:1/18:0) | 22 | 10 |
| NSM | SM(d18:1/24:1) | 22 | 10 |
| CMH | GlcCer(d18:1/16:0) | 8 | 0 |
| CHL | Cholesterol | 70 | 58 |

**References**

15. Jensen, O., Brockmöller, J., and Dücker, C. (2021) Identification of Novel High-Affinity Substrates of OCT1 Using Machine Learning-Guided Virtual Screening and Experimental Validation. J Med Chem. 10.1021/acs.jmedchem.0c02047 https://doi.org/10.1021/acs.jmedchem.0c02047

20. Wittern, C. I., Schröder, S., Jensen, O., Brockmöller, J., and Gebauer, L. (2024) Comprehensive characterization of the OCT1 phenylalanine-244-alanine substitution reveals highly substrate-dependent effects on transporter function. J Biol Chem. 300, 107835 https://doi.org/10.1016/j.jbc.2024.107835

22. Meyer, M. J., Neumann, V. E., Friesacher, H. R., Zdrazil, B., Brockmöller, J., and Tzvetkov, M. V. (2019) Opioids as Substrates and Inhibitors of the Genetically Highly Variable Organic Cation Transporter OCT1. *J Med Chem*. **62**, 9890–9905 https://doi.org/10.1021/acs.jmedchem.9b01301

23. Meyer, M. J., Schreier, P. C. F., Basaran, M., Vlasova, S., Seitz, T., Brockmöller, J., Zdrazil, B., and Tzvetkov, M. V. (2022) Amino acids in transmembrane helix 1 confer major functional differences between human and mouse orthologs of the polyspecific membrane transporter OCT1. J Biol Chem. 10.1016/j.jbc.2022.101974 https://doi.org/10.1016/j.jbc.2022.101974

24. Römer, S., Lazzarin, E., Neumann, A., Lindemann, E., Meyer-Tönnies, M. J., Stockner, T., and Tzvetkov, M. V. (2025) Substrate-specific effects point to the important role of Y361 as part of the YER motif in closing the binding pocket of OCT1. *Journal of Biological Chemistry*. 10.1016/j.jbc.2025.108318 https://doi.org/10.1016/j.jbc.2025.108318

27. Zhang, S., Zhu, A., Kong, F., Chen, J., Lan, B., He, G., Gao, K., Cheng, L., Sun, X., Yan, C., Chen, L., and Liu, X. (2024) Structural insights into human organic cation transporter 1 transport and inhibition. *Cell Discov*. **10**, 30 https://doi.org/10.1038/s41421-024-00664-1

54. Pogozheva, I. D., Armstrong, G. A., Kong, L., Hartnagel, T. J., Carpino, C. A., Gee, S. E., Picarello, D. M., Rubin, A. S., Lee, J., Park, S., Lomize, A. L., and Im, W. (2022) Comparative Molecular Dynamics Simulation Studies of Realistic Eukaryotic, Prokaryotic, and Archaeal Membranes. *J. Chem. Inf. Model.* **62**, 1036–1051 https://doi.org/10.1021/acs.jcim.1c01514

60. Meyer, M. J., Tuerkova, A., Römer, S., Wenzel, C., Seitz, T., Gaedcke, J., Oswald, S., Brockmöller, J., Zdrazil, B., and Tzvetkov, M. V. (2020) Differences in Metformin and Thiamine Uptake between Human and Mouse Organic Cation Transporter 1: Structural Determinants and Potential Consequences for Intrahepatic Concentrations. *Drug Metab Dispos*. **48**, 1380–1392 https://doi.org/10.1124/dmd.120.000170

61. Kim, S., Chen, J., Cheng, T., Gindulyte, A., He, J., He, S., Li, Q., Shoemaker, B. A., Thiessen, P. A., Yu, B., Zaslavsky, L., Zhang, J., and Bolton, E. E. (2025) PubChem 2025 update. *Nucleic Acids Research*. **53**, D1516–D1525 https://doi.org/10.1093/nar/gkae1059

62. Dos Santos Pereira, J. N., Tadjerpisheh, S., Abu Abed, M., Saadatmand, A. R., Weksler, B., Romero, I. A., Couraud, P.-O., Brockmöller, J., and Tzvetkov, M. V. (2014) The poorly membrane permeable antipsychotic drugs amisulpride and sulpiride are substrates of the organic cation transporters from the SLC22 family. *AAPS J*. **16**, 1247–1258 https://doi.org/10.1208/s12248-014-9649-9

63. Meyer, M. J., Seitz, T., Brockmöller, J., and Tzvetkov, M. V. (2017) Effects of genetic polymorphisms on the OCT1 and OCT2-mediated uptake of ranitidine. *PLoS ONE*. **12**, 0189521 https://doi.org/10.1371/journal.pone.0189521

64. Varadi, M., Anyango, S., Deshpande, M., Nair, S., Natassia, C., Yordanova, G., Yuan, D., Stroe, O., Wood, G., Laydon, A., Žídek, A., Green, T., Tunyasuvunakool, K., Petersen, S., Jumper, J., Clancy, E., Green, R., Vora, A., Lutfi, M., Figurnov, M., Cowie, A., Hobbs, N., Kohli, P., Kleywegt, G., Birney, E., Hassabis, D., and Velankar, S. (2021) AlphaFold Protein Structure Database: massively expanding the structural coverage of protein-sequence space with high-accuracy models. *Nucleic Acids Res*. 10.1093/nar/gkab1061 https://doi.org/10.1093/nar/gkab1061
